# Growing Sodiophilic ZnO Nanorod Arrays on Al Substrate for High-Energy-Density Anode-Free Na Batteries

**DOI:** 10.1021/jacs.5c16420

**Published:** 2025-12-04

**Authors:** Yongling An, Zhihao Pei, Deyan Luan, Xiong Wen David Lou

**Affiliations:** Department of Chemistry, 53025 City University of Hong Kong, 83 Tat Chee Avenue, Kowloon, Hong Kong 999077, China

## Abstract

Anode-free Na batteries maximize energy density but suffer from poor cycling performance originating from the uncontrolled Na growth and large volume change. Herein, we report dense ZnO nanorod arrays on Al foil (D-ZnO NRAs/Al) synthesized by electrodeposition and low-temperature heat treatment methods as a lightweight current collector for selective nucleation and homogeneous plating of Na. The nanorod arrays not only offer abundant interspace to reduce structural stress and enable high Na plating but also promote uniform Na deposition by manipulating Na^+^ ion flux and reducing local current density. In addition, the sufficient sodiophilic active sites minimize nucleation barriers and serve as preferred nucleation sites, thereby homogenizing Na nucleation and growth. As expected, the D-ZnO NRAs/Al host presents a highly reversible Na plating/stripping with low nucleation overpotential and dendrite-free Na morphology. When coupled with a 4.3 V-class cathode, the assembled anode-free pouch cell achieves stable cycling performance with a capacity retention of 86% for 105 cycles and a high energy density of 441.7 Wh kg^–1^ based on the active materials of both anode and cathode.

## Graphical abstract

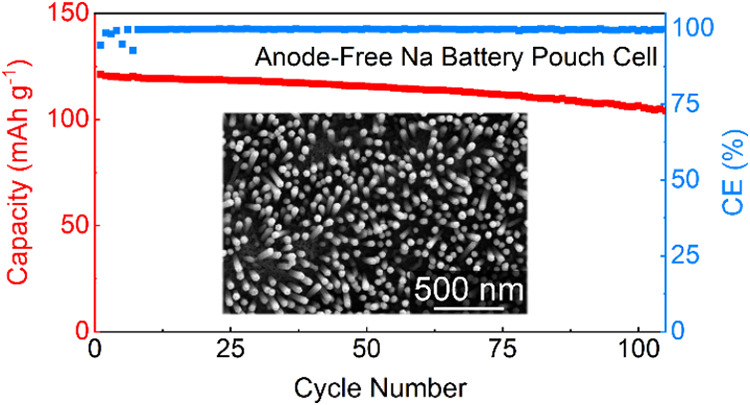

## 1 Introduction

Considering the abundant reserves and global distributions of Na, Na-ion batteries (NIBs) have been regarded as economical technologies for electrical energy storage.^1,2^ However, the relatively large atomic weight and size of Na limit the energy density of NIBs.^3,4^ An anode-free configuration, built by coupling a sodiumized cathode with a bare current collector, can maximize energy density and minimize costs synchronously.^5,6^ However, realizing stable cycling performance in anode-free Na batteries (AFNBs) remains challenging because of the finite Na source and the irreversible consumption of Na^+^ ions.^7,8^ Achieving stable and uniform Na deposition/dissolution with high Coulombic efficiency (CE) is vital for enhancing the capability of AFNBs.^9,10^

Regulating Na nucleation, plating, and stripping can enhance the stability of electrochemically deposited Na.^11,12^ The current collector plays a crucial role in Na nucleation and growth, directly deciding cycling performance.^13,14^ Constructing an artificial interlayer with sodiophilic materials on sodiophobic current collectors can effectively promote homogeneous Na plating and inhibit dendritic formation.^15,16^ However, micron-scale interface layers typically exhibit high nucleation overpotentials, which inevitably couple with poor Na^+^ ion transport and are unable to meet the requirements for high-rate performance.^17,18^ Additionally, these interface layers are easily destroyed by the large volume changes originating from Na deposition/dissolution at high current densities and depth of discharge (DOD).^19,20^ Therefore, designing an advanced microinterface architecture is vital for inhibiting the formation of Na dendrites and achieving long-term operation of AFNBs at high rates.^21,22^

Three-dimensional (3D) conductive skeletons (such as carbon fiber,^5,23^ porous carbon,^10,24^ porous fluorinated framework,^20^ porous Al^25^) that function as current collectors are pivotal for improving the performance of cells. Compared to planar electrodes, the designed 3D porous frameworks with high surface areas can promote uniform Na plating by reducing local current density and manipulating the distribution of Na^+^ ion flux.^26,27^ Besides, the 3D open interface provides abundant inner space to reduce structural stress and confine Na plating.^20,28^ The synergistic integration of hierarchical porosity, high electrical conductivity, and good sodiophilicity offers the potential for 3D substrates to boost the performance of AFNBs.^10,24^ Despite delightful progress, dendritic Na growth and segregation problems are still detected on the surface of these current collectors, further deteriorating the cycling performance of AFNBs.^17,19^ Besides, the weak bonding interaction between Na and the matrix promotes defect and crack formation.^29,30^ A large amount of Na is plated on the upper surface of the 3D architecture, resulting in inefficient space utilization.^25,31^ Consequently, it is urgent to restructure 3D conductive skeletons to enable uniform Na growth while sustaining reversible Na electrochemical reactions.^17,19,32^ Regarding topological engineering, the in situ fabricated 3D array architectures can prevent direct contact between the electrolyte and substrate, thus enhancing electrochemical stability at the anode-electrolyte interface.^33^ The enlarged interfacial area further offers ideal environments for the formation of affluent nucleation sites, promoting homogeneous Na nucleation and growth.^34^ Besides, the 3D array structure can improve structural stability, accommodate volume change, facilitate rapid ion transport by eliminating binders and conductive agents.^35^

In this work, dense ZnO nanorod arrays are in situ grown on lightweight Al foil (D-ZnO NRAs/Al) by electrodeposition and low-temperature heat treatment methods as a current collector for AFNBs. Benefiting from the array structures, the D-ZnO NRAs/Al can not only promote uniform Na growth by regulating Na^+^ ion flux and reducing the local current density but also provide enough interspace to reduce structural stress and tightly confine Na deposition. Besides, the ZnO nanorods with abundant sodiophilic sites can minimize nucleation barriers and function as preferred nucleation sites, subsequently manipulating homogeneous Na nucleation and growth. Thanks to these favorable merits, the D-ZnO NRAs/Al host exhibits an ultrahigh CE of about 100% at 10 mA cm^–2^ and 20 mAh cm^–2^. Moreover, the D-ZnO NRAs/Al–Na anode can operate stably for 2000 h at 10 mA cm^–2^ and 10 mAh cm^–2^ (DOD = 50%). Furthermore, a proof-of-concept anode-free pouch cell with a high-voltage Na_3_V_2_O_2_(PO_4_)_2_F (NVOPF) cathode and a D-ZnO NRAs/Al host delivers a high energy density of 441.7 Wh kg^–1^ (based on the active materials of both the anode and cathode) and stable cycling performance with 86.0% capacity retention after 105 cycles.

## 2 Results and Discussion

### 2.1 Fabrication and Characterization of D-ZnO NRAs/Al

D-ZnO NRAs/Al is synthesized by electrodeposition and low-temperature heat treatment methods (Figure 1A). Initially, Zn nanosheet arrays are electrodeposited on the surface of Al foil (Zn NSAs/Al). Figures S1 and S2 in Supporting Information show field-emission scanning electron microscope (FESEM) images and X-ray diffraction (XRD) pattern of the Al foil. By the electrodeposition method, uniform Zn nanosheet arrays are grown on the Al foil surface (Figures 1B,C, and S3). XRD pattern displays the characteristic peaks of both Al and Zn (Figure S4), implying successful plating of Zn on the Al foil.

**Figure 1:**
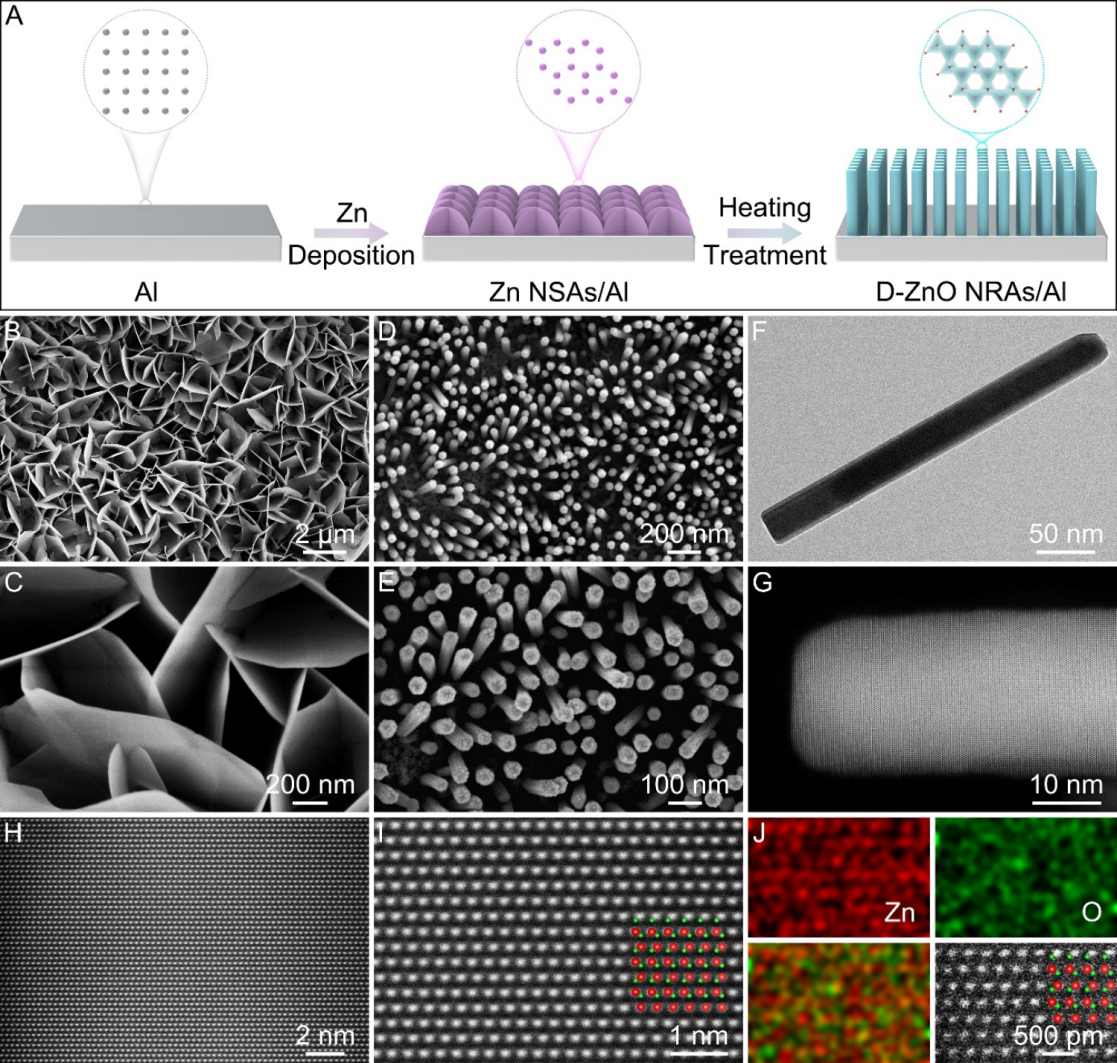
Morphological characterization of Zn NSAs/Al and D-ZnO NRAs/Al. (A) Schematic illustration of the preparation for D-ZnO NRAs/Al. (B, C) FESEM images of Zn NSAs/Al. (D, E) FESEM, (F) TEM, (G–I) HAADF-STEM, and (J) elemental mapping images of D-ZnO NRAs/Al.

After the subsequent low-temperature heat treatment process, the Zn nanosheet arrays are converted into dense ZnO nanorod arrays on the Al foil surface (D-ZnO NRAs/Al). The obtained D-ZnO NRAs/Al preserve homogeneous and dense nanorod arrays (Figure 1D,E). These arrays are favorable for decreasing the local current density, offering void space to confine Na plating, and increasing the contact area between the electrodes and electrolytes to promote ion diffusion. Transmission electron microscopy (TEM) image discloses the nanorod structure with a diameter of 32.6 nm and a length of 325.9 nm (Figure 1F). The high-angle annular dark-field scanning TEM (HAADF-STEM) method is used to investigate the structure of D-ZnO NRAs/Al. HAADF-STEM image of D-ZnO NRAs/Al illustrates that the entire nanorod exhibits excellent crystallinity (Figure 1G). Besides, clear lattice fringes without dislocations or stacking faults are observed (Figures 1H, I, and S5), which align well with the ZnO model. Elemental mapping images show a homogeneous distribution of Zn and O elements in the D-ZnO NRAs/Al (Figure 1J).

By simply adjusting the treatment temperature, the distributed density of ZnO nanorod arrays on Al foil can be controlled (Figures 2 and S6). At a treatment temperature of 440 °C, sparse ZnO nanorod arrays are grown on the Al foil surface (S-ZnO NRAs/Al, Figure 2A,E). When the treatment temperature increases to 460 °C, the product exhibits a denser distribution of ZnO nanorods (Figure 2B,F). Further increasing the treatment temperature to 480 °C leads to an improvement in the distribution density of ZnO nanorods (Figure 2C,G). When the treatment temperature reaches 500 °C, the original Zn nanosheet arrays evolve into dense and homogeneous ZnO nanorod arrays (Figure 2D,H). As shown in Figures 2I–L and S7, the products synthesized at different temperatures exhibit a single-crystal structure throughout the entire nanorod. Besides, the Zn and O elements are uniformly distributed on the nanorods in all the products (Figure 2M–P). XRD patterns show characteristic peaks for both ZnO and Al in the products synthesized at different temperatures (Figure S8), which align with the results of energy dispersive X-ray (EDX) (Figure S9). Therefore, single-crystal ZnO nanorod arrays with varying distribution densities are successfully grown on the Al foil surface. The distributed density of ZnO nanorod arrays can be manipulated by adjusting the temperature during the heat treatment process. In contrast to other hosts, Al foil is more favorable in terms of weight and can serve as a current collector for both electrodes in Na batteries because it does not undergo alloying with Na metal (Figure S10).

**Figure 2:**
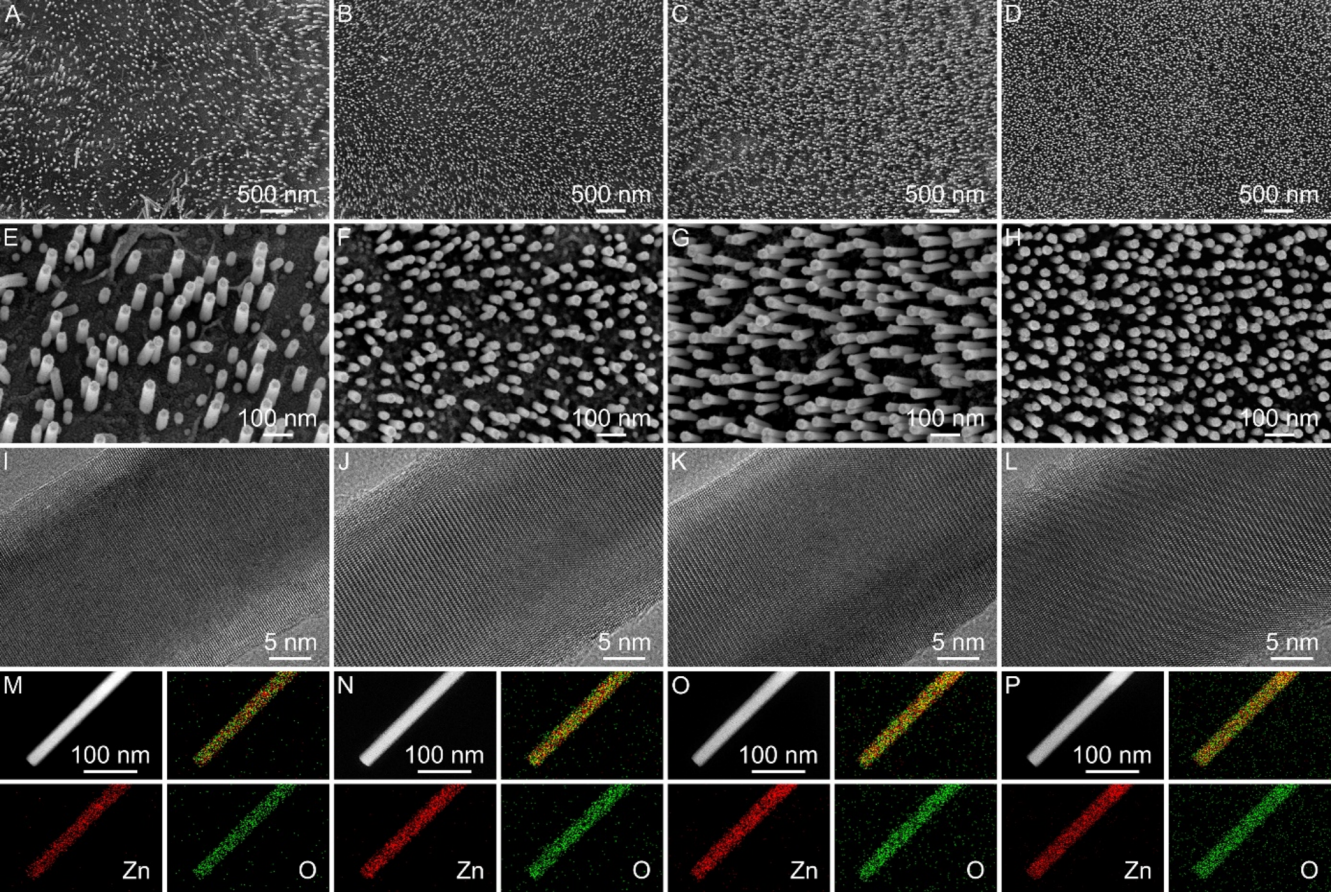
Morphological evolution of ZnO NRAs/Al. (A–H) FESEM, (I–L) TEM, (M–P) HAADF-STEM and elemental mapping images of ZnO NRAs/Al synthesized at different temperatures of (A, E, I, M) 440 °C, (B, F, J, N) 460 °C, (C, G, K, O) 480 °C, and (D, H, L, P) 500 °C.

### 2.2 Theoretical Calculation and Na Plating Behavior

Density functional theory (DFT) computations elucidate the contribution of each component in D-ZnO NRAs/Al (Figures S11 and S12). Sodiophilicity denotes the ability to adsorb and bond Na atoms, which can decrease the energy barrier for Na nucleation.^5,6^ The binding energy between Na and the host is suggested as a quantitative measure of sodiophilicity. The NaZn_13_ exhibits high binding energy (Figure 3A),^14^ implying the sodiophilic nature of NaZn_13_. Besides, DFT-based interfacial charge density results further disclose the strong interaction between Na atoms and NaZn_13_, with obvious charge transfer at the interface (Figure 3B). Benefiting from this strong affinity, the aggregation of plated Na in the D-ZnO NRAs/Al can be mitigated to avoid the formation of Na dendrites.

**Figure 3:**
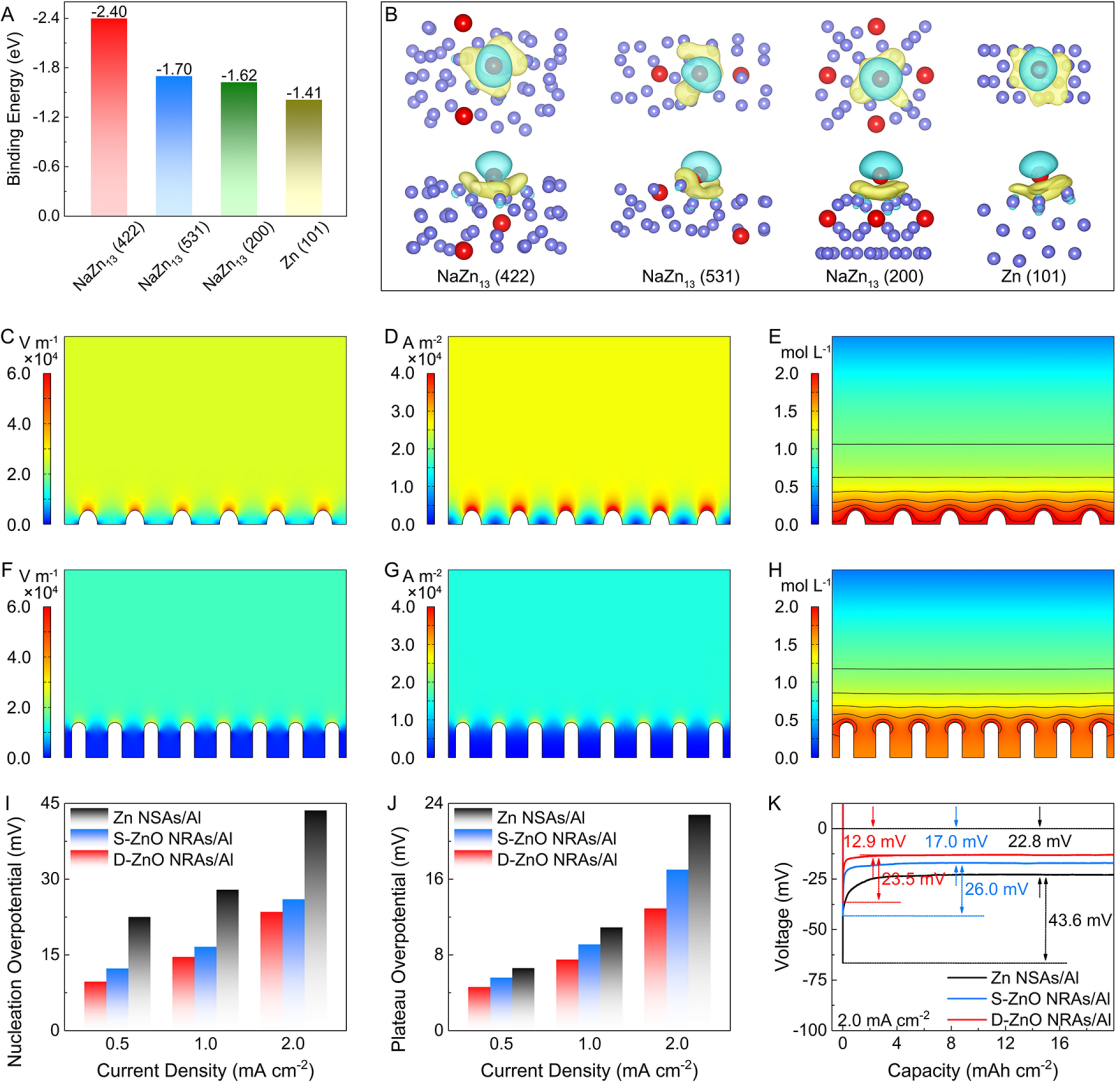
Theoretical and electrochemical results of D-ZnO NRAs/Al host. (A) Summary of the calculated binding energy of Na atoms with NaZn_13_ and Zn. (B) Interfacial charge-density models of NaZn_13_ and Zn with Na atom adsorption. Simulated distributions of (C, F) electric field, (D, G) current density, and (E, H) electrolyte concentration on (C–E) Al and (F–H) D-ZnO NRAs/Al. (I) Nucleation and (J) plateau overpotentials of different hosts tested at current densities of 0.5, 1.0, and 2.0 mA cm^–2^. (K) Voltage-capacity curves during Na nucleation on different hosts tested at 2.0 mA cm^–2^.

Finite element simulations are conducted to visualize the electric field intensity, current density, and electrolyte concentration in the D-ZnO NRAs/Al. As presented in Figure 3C, regions with “hot spots” describing obviously reinforced electric fields are detected at the hump surface of the Al foil, exacerbating the formation of Na dendrites. Besides, inhomogeneous current density and electrolyte concentration are observed on the Al substrate (Figure 3D,E), indicating the possibility of fast dendrite growth. In contrast, a uniform distribution of electric field intensity during the electroplating process is detected for the D-ZnO NRAs/Al host (Figure 3F), which can be ascribed to the preferred Na plating among the nanorod arrays. As the Na^+^ ion flux flows into the interior of the D-ZnO NRAs/Al, Na plating occurs directly on the surface of sodiophilic components, resulting in the dispersion of ion flux and the reduction of current density in the channels with large plating areas (Figure 3G). Moreover, the D-ZnO NRAs/Al can also uniformize the distribution of electrolyte concentration and offer abundant contact between the electrode and electrolyte (Figure 3H). Therefore, the simulation results indicate that the D-ZnO NRAs/Al host is anticipated to homogenize the distribution of electric field intensity, current density, and electrolyte concentration, enabling long-term cycling performance of batteries.

The initial nucleation stage critically governs the quality of Na plating.^36,37^ The nucleation and plateau overpotentials are characterized at different current densities. The D-ZnO NRAs/Al host displays the lowest Zn nucleation overpotential compared with other electrodes at different current densities (Figures 3I and S13), suggesting a reduction in Na nucleation barriers on the D-ZnO NRAs/Al due to the abundant sodiophilic sites. Moreover, the plateau overpotential of the D-ZnO NRAs/Al host is also lower compared to other hosts (Figures 3J and S13), implying rapid Na^+^ ion migration to the host surface. For instance, the nucleation and plateau overpotentials of the D-ZnO NRAs/Al host are 23.5 mV and 12.9 mV, respectively, which are lower than those of Zn NSAs/Al and S-ZnO NRAs/Al (Figure 3K). The functional sodiophilic ZnO sites can reduce nucleation barriers and function as preferred nucleation sites, thus manipulating homogeneous Na growth. Besides, the array structure greatly improves the contact area between the electrolytes and electrodes, thus facilitating electrolyte penetration and ion diffusion.

To validate the superiority of the D-ZnO NRAs/Al host, the Na deposition behavior on different hosts is characterized (Figure S14). At a plating capacity of 20 mAh cm^–2^, large Na dendrites are formed on the surface of the Zn NSAs/Al host, resulting in potential safety problems. In comparison, the Na plating behavior on the S-ZnO NRAs/Al and D-ZnO NRAs/Al hosts is greatly improved. For the S-ZnO NRAs/Al host, the formation of Na dendrites is suppressed. Impressively, a dense and smooth surface without any obvious Na dendrites is achieved for the D-ZnO NRAs/Al host. The excellent regulation of Na deposition in the D-ZnO NRAs/Al host is attributed to the abundant sodiophilic sites and nanorod array structure. The high-affinity Na binding sites of ZnO nanorods minimize nucleation barriers and serve as preferred nucleation sites, further homogenizing Na nucleation and plating. The nanorod array structure can not only uniformize ion flux distribution, decrease local current density, and provide void space to confine Na deposition but also improve the contact area between the electrodes and electrolytes to promote ion diffusion, thereby realizing homogeneous Na plating and high deposition capacity.

### 2.3 Electrochemical Performance Evaluation

The reversibility of Na plating/stripping, which is significant for functionality in an anode-free configuration, is evaluated by CE. Compared to that of the Zn NSAs/Al and S-ZnO NRAs/Al hosts, the onset potential of Na deposition/dissolution on the D-ZnO NRAs/Al host is the lowest (Figures 4A and S15). In addition, the large closed area and high current density reveal a greater number of active nucleation sites and a larger contact area between the electrodes and electrolytes, implying rapid electrochemical reaction kinetics.^5^ As shown in Figures 4B and S16, the D-ZnO NRAs/Al host displays improved reversibility and cycling stability with a high average CE of 99.95% at 1 mA cm^–2^ and 1 mAh cm^–2^ for 1500 cycles. In comparison, the Zn NSAs/Al host exhibits a low average CE of 99.55% for 200 cycles with obvious fluctuations, and the S-ZnO NRAs/Al host suffers from severe fluctuations after only 760 cycles. The corresponding plating/stripping voltage curves exhibit that the voltage hysteresis of the D-ZnO NRAs/Al host is only 16.6 mV, lower than those of the Zn NSAs/Al (27.1 mV) and S-ZnO NRAs/Al (17.4 mV), which indicates the fast reaction kinetics of the D-ZnO NRAs/Al (Figure 4C and Table S1), consistent with the electrochemical impedance spectroscopy (EIS) results (Figure S17). Compared to the Zn NSAs/Al and S-ZnO NRAs/Al hosts, a dense surface of the D-ZnO NRAs/Al host is obtained after cycling (Figure S18). Besides, the effectiveness of the D-ZnO NRAs/Al host is further verified by CE measurements at higher current densities and areal capacities. At an areal capacity of 1 mAh cm^–2^, the D-ZnO NRAs/Al host realizes high average CEs of 99.97%, 99.97%, and 99.96% at 2, 5 and 8 mA cm^–2^, respectively (Figure 4D and Table S2). Furthermore, the CE of the D-ZnO NRAs/Al host tested at different areal capacities of 1, 2 and 5 mAh cm^–2^ with a current density of 10 mA cm^–2^ is explored (Figure 4E). The average CEs are around 99.95%, 99.96%, and 99.93%. The corresponding charge and discharge profiles for the D-ZnO NRAs/Al host are displayed in Figure S19. Moreover, the areal capacity is continuously increased from 8 to 20 mAh cm^–2^ (Figures S20, S21, and 4F). For instance, stable voltage curves and high electrode reversibility (CE ≈ 100%) are observed at 10 mA cm^–2^ and 20 mAh cm^–2^. The performance of the D-ZnO NRAs/Al host surpasses that of most previously reported hosts (Table S3).

**Figure 4:**
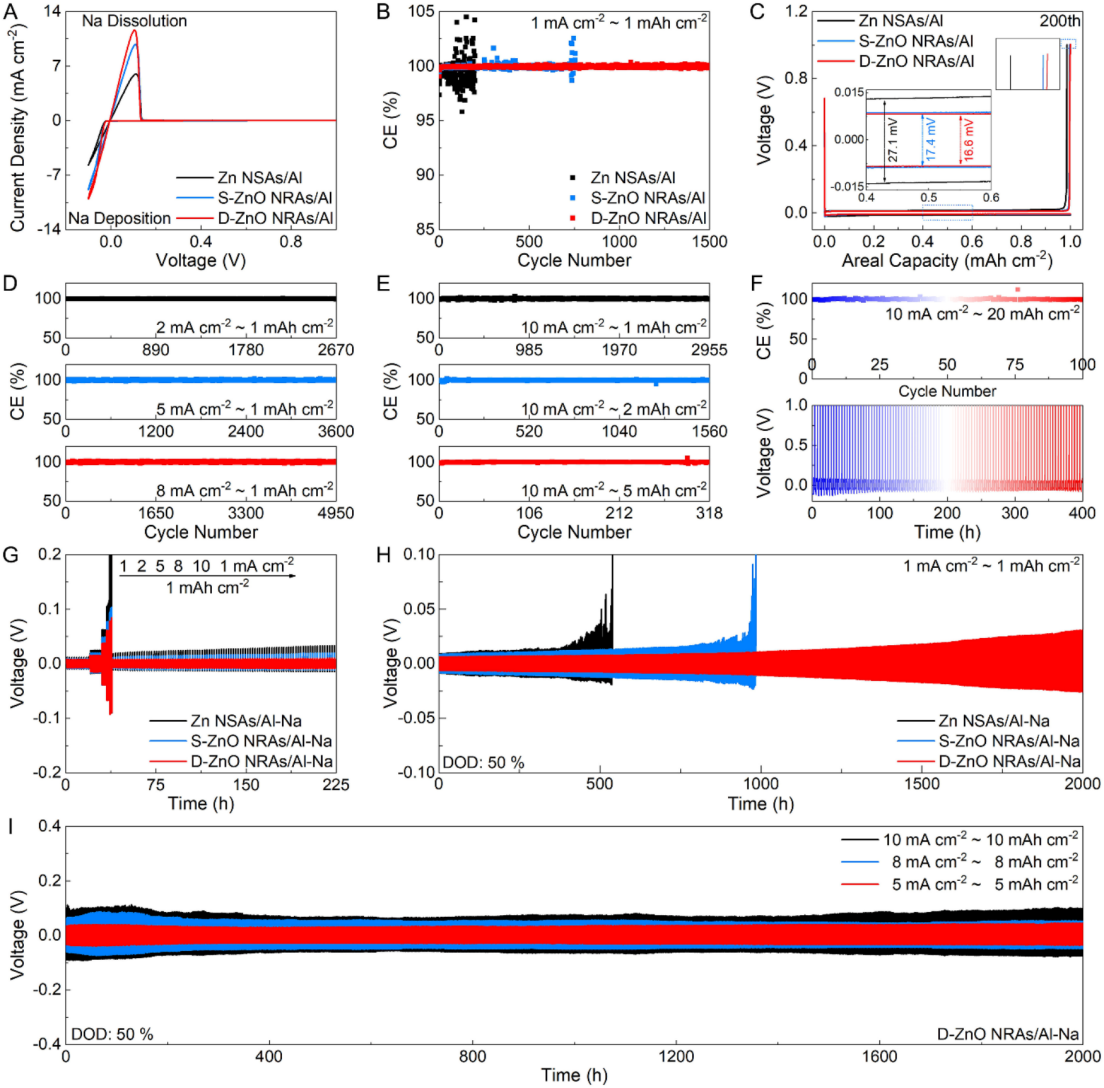
Electrochemical performance of D-ZnO NRAs/Al host. (A) CV curves, (B) CE plots, and (C) plating/stripping voltage curves at the 200th cycle of different hosts. (D, E) CE plots of D-ZnO NRAs/Al host tested at different (D) current densities and (E) areal capacities. (F) CE plot and voltage–time profiles of D-ZnO NRAs/Al host tested at 10 mA cm^–2^ and 20 mAh cm^–2^. (G) Rate capability and (H) cycling performance of different anodes. (I) Cycling performance of D-ZnO NRAs/Al–Na anode tested at different current densities and areal capacities.

In addition, the cycling performance of the Zn NSAs/Al–Na, S-ZnO NRAs/Al–Na, and D-ZnO NRAs/Al–Na anodes is investigated. The D-ZnO NRAs/Al–Na anode delivers good rate capability with improved voltage hysteresis of 15.2, 30.6, 76.4, 127.9, and 170.1 mV as the current density improves from 1 to 10 mA cm^–2^ (Figure 4G). By contrast, the Zn NSAs/Al–Na and S-ZnO NRAs/Al–Na anodes exhibit higher voltage hysteresis. Besides, the D-ZnO NRAs/Al–Na anode displays excellent long-term cycling performance with a gradually increased voltage hysteresis over 2000 h at 1 mA cm^–2^ and 1 mAh cm^–2^ (Figure 4H and Table S4). In comparison, the S-ZnO NRAs/Al–Na anode suffers from serious polarization after 985 h, and the Zn NSAs/Al–Na anode can hardly operate for 539 h (Figure S22). This may be attributed to the internal short circuit induced by the Na dendrite growth and the “dead Na” accumulation at the anode interface.^37,38^ FESEM images show a compact and smooth surface with dendrite-free morphology for the D-ZnO NRAs/Al–Na anode after cycling (Figure S23). In general, high current density and DOD exacerbate the Na dendrite growth and volume expansion, leading to a short cycle life.^7,20^ At a DOD value of 50%, the D-ZnO NRAs/Al–Na anode shows superior cycling performance with consistent voltage hysteresis over 2000 h at high current densities and areal capacities (Figures 4I, S24, and S25). Even when tested at high DOD values of 60%, 70%, and 80%, the D-ZnO NRAs/Al–Na anode can still realize a long lifespan (Figures S26–S28), again implying the superior reversibility and durability of the D-ZnO NRAs/Al–Na anode. The excellent cycling performance of the D-ZnO NRAs/Al–Na anode is comparable with that of most composite Na anodes on various hosts reported previously (Table S5).

To further verify the feasibility of the D-ZnO NRAs/Al, anode-less and anode-free batteries are constructed using an NVOPF cathode (Figures S29 and S30). The electrochemical performance of the Na//NVOPF cell is probed (Figures S31–S33). Compared with the Zn NSAs/Al–Na//NVOPF and S-ZnO NRAs/Al–Na//NVOPF cells, the D-ZnO NRAs/Al–Na//NVOPF cell shows smaller voltage polarization in the CV curves (*N*/P = 1.5, Figures 5A and S34), indicating the rapid reaction kinetics endowed by the D-ZnO NRAs/Al–Na anode. In addition, the D-ZnO NRAs/Al–Na//NVOPF cell tested at 1C displays cycling performance with a capacity retention of 95.2% over 150 cycles (*N*/P = 1.5, Figure 5B). In comparison, the Zn NSAs/Al–Na//NVOPF and S-ZnO NRAs/Al–Na//NVOPF cells experience failure after only 53 cycles and 92 cycles, respectively (Figure S35). The improved performance is ascribed to the highly reversible Na deposition/dissolution with dendrite-free morphology (Figure S36). Even at a higher current density of 2C (*N*/P = 1.5), the D-ZnO NRAs/Al–Na//NVOPF cell achieves stable cycling performance with a capacity retention of 91.4% over 300 cycles (Figures 5C and S37). The rate capabilities of three full cells are compared (*N*/P = 1.5, Figure 5D), where the D-ZnO NRAs/Al–Na//NVOPF cell delivers high capacities at different current densities ranging from 0.5C to 10C (Figure S38), indicating improved rate capability. The EIS results reveal a small charge transfer resistance for the D-ZnO NRAs/Al–Na//NVOPF cell (Figure S39), which rationalizes the enhanced rate performance. Besides, the cycling performance of the D-ZnO NRAs/Al–Na//NVOPF cell tested at different N/P ratios and mass loadings of the NVOPF cathode is investigated. At a low N/P ratio of 1.0, the D-ZnO NRAs/Al–Na//NVOPF cell achieves stable cycling performance with a capacity retention of 94.7% after 120 cycles (Figures 5E and S40). When the N/P ratio increases from 2.0 to 4.0, the D-ZnO NRAs/Al–Na//NVOPF cell still presents favorable durability (Figure S41). Although a high N/P ratio can boost cycling stability, it inevitably compromises the energy density of the batteries. Optimizing this trade-off is key for the development of anode-less batteries. In addition, at high mass loadings ranging from 9.43 mg cm^–2^ to 32.62 mg cm^–2^ (Figure S42), the D-ZnO NRAs/Al–Na//NVOPF cell can still operate stably for 105 cycles (Figures 5F and S43). Inspired by the excellent capability of coin cell, a pouch cell is constructed to investigate the practicability of the D-ZnO NRAs/Al–Na//NVOPF cell. As shown in Figures 5G and S44, a stable cycling performance with a capacity retention of 88.9% for 105 cycles at 1C is obtained for the D-ZnO NRAs/Al–Na||NVOPF pouch cell (*N*/P = 1.5). The D-ZnO NRAs/Al–Na||NVOPF cell demonstrates superior electrochemical performance compared to state-of-the-art anode-less Na batteries reported previously (Table S6). By minimizing the volume and weight of batteries, the anode-free architecture maximizes energy density.^39,40^ The assembled anode-free D-ZnO NRAs/Al||NVOPF pouch cell delivers stable cycling performance with a capacity of 104.2 mAh g^–1^ and a capacity decay of 0.1333% per cycle over 105 cycles (Figures 5H and S45). Besides, a high energy density of 441.7 Wh kg^–1^ is realized for this anode-free pouch cell based on the active materials of both anode and cathode. The cycling performance of the anode-free D-ZnO NRAs/Al||NVOPF pouch cell surpasses that of most AFNBs previously reported (Table S7). After cycling, the morphology of D-ZnO NRAs/Al remain stable (Figure S46). More importantly, this anode-free pouch cell can power a phone, an LED light, an LED sign, and a rotating fan (Figures 5I and S47), validating its inherent safety in practical operating environments.

**Figure 5:**
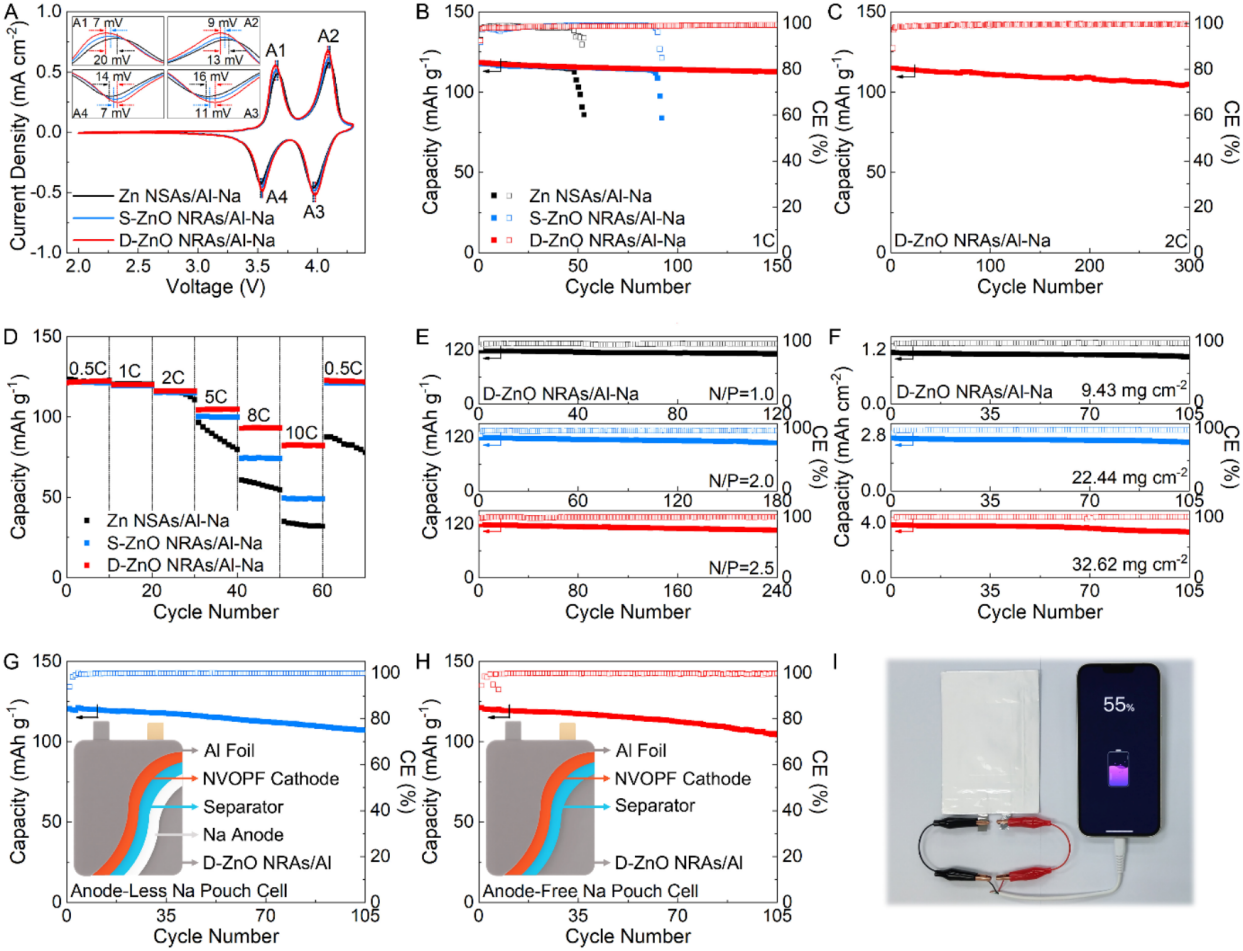
Electrochemical performance of anode-less and anode-free Na batteries. (A) CV curves and (B) cycling performance of different full cells (*N*/P = 1.5). (C) Cycling performance of D-ZnO NRAs/Al–Na//NVOPF cell tested at 2C (*N*/P = 1.5). (D) Rate capability of different full cells (*N*/P = 1.5). (E, F) Cycling performance of D-ZnO NRAs/Al–Na//NVOPF cell with different (E) *N*/P ratios and (F) NVOPF loadings. (G, H) Cycling performance of (G) D-ZnO NRAs/Al–Na//NVOPF pouch cell (N/*P* = 1.5) and (H) D-ZnO NRAs/Al//NVOPF pouch cell (*N*/P = 0). (I) Optical image of a fully charged D-ZnO NRAs/Al//NVOPF pouch cell used to light up a phone.

## 3 Conclusion

In summary, D-ZnO NRAs/Al is rationally designed as a multifunctional host composed of well-organized ZnO nanorod array structures and abundant sodiophilic sites to enable high-energy-density AFNBs. The nanorod arrays can not only uniformize Na plating by reducing the local current density and controlling Na^+^ ion flux but also offer abundant interspace to relieve structural stress and achieve high Na plating. Besides, the ZnO nanorods provide initial sodiophilic sites to minimize nucleation barriers, further promoting uniform Na nucleation and growth. As a result, the D-ZnO NRAs/Al host can achieve highly reversible Na plating/stripping with a high average CE of 100% at 10 mA cm^–2^ and 20 mAh cm^–2^. Besides, the D-ZnO NRAs/Al–Na anode can operate stably for 2000 h at 10 mA cm^–2^ and 10 mAh cm^–2^ with a DOD value of 50%. Furthermore, the assembled anode-less Na pouch cell displays stable cycling performance with a capacity retention of 88.9% for 105 cycles (*N*/P = 1.5). More importantly, a 4.3 V-class anode-free pouch cell is constructed, achieving a high energy density of 441.7 Wh kg^–1^ based on the active materials of both anode and cathode. This work opens up possibilities for developing energy-dense batteries through rationally engineered host architectures.

## Supplementary Material



## Notes

Experimental and computational details, additional structural and morphological characterizations, including XRD, EDX, FESEM, HRTEM, and DFT calculations results (PDF)
