# Supplementary material for: Growing Sodiophilic ZnO Nanorod Arrays on Al Substrate for High-Energy-Density Anode-Free Na Batteries
Source: J Am Chem Soc. 2025 Dec 4;147(50):46440–8. doi: 10.1021/jacs.5c16420 (PMC12715786; doi:10.1021/jacs.5c16420)
Supplement: Supplementary file 1 [file ja5c16420_si_001.pdf]

Supporting Information for

Growing Sodiophilic ZnO Nanorod Arrays on Al Substrate for High-Energy-Density Anode-Free Na Batteries

Yongling An,[†] Zhihao Pei,[†] Deyan Luan,[†] Xiong Wen (David) Lou[†]*

[†]Department of Chemistry, City University of Hong Kong, 83 Tat Chee Avenue, Kowloon, Hong Kong, 999077 China

*Corresponding author. Email: david.lou@cityu.edu.hk

MATERIALS AND METHODS

Synthesis of Zn NSAs/Al

Zn NSAs/Al was synthesized by an electrodeposition approach. An Al foil treated with ethanol and deionized water was used as the working electrode, while a Zn foil was used as both counter electrode and reference electrode. The electrodeposition bath was prepared by mixing 4 mg of cetyltrimethylammonium bromide, 13 mg of $(NH_4)_2SO_4$, and 172 mg of $ZnSO_4 \cdot 7H_2O$ in 100 mL of deionized water. Zn nanosheets were plated on the Al foil surface at a current density of 1 mA cm^{-2} for 1 h. The resultant Al-Zn substrate was then rinsed with deionized water for subsequent use.

Synthesis of D-ZnO NRAs/Al

D-ZnO NRAs/Al was synthesized by a low-temperature heating treatment process from the Zn NSAs/Al. In detail, the as-obtained Zn NSAs/Al was heated at 500 °C for 24 h in an Ar/H_2 atmosphere.

Synthesis of S-ZnO NRAs/Al

S-ZnO NRAs/Al was synthesized through a similar procedure as D-ZnO NRAs/Al, except that a temperature of 440 °C was used for the heating treatment process.

Materials characterizations

The crystal phase of the products was collected by X-ray diffraction (XRD) using a Bruker D2 Phaser X-ray diffractometer. The structure and morphology of the products were analyzed by transmission electron microscope (TEM, JEOL JEM-2100) and field-emission scanning electron microscopy (FESEM, JSM-7800F). Elemental mapping image was characterized by TEM equipped with energy-dispersive X-ray (EDX) spectroscopy. The compositions were evaluated using EDX spectroscopy linked to the FESEM and TEM instruments.

Electrochemical measurements

Electrochemical measurements were evaluated on a NEWARE testing system. Electrochemical impedance spectroscopy (EIS) was conducted on an electrochemical workstation (CHI 660E) from 100 kHz to 10 MHz. Cyclic voltammetry (CV) was also characterized on the same workstation. Glass fiber was used as a separator, and 1 M $NaPF_6$ in diglyme was used as an electrolyte. The electrolyte volume was approximately 80 μL per cell. To probe the Coulombic efficiency (CE) of Na deposition/dissolution, Na foil was used both as the counter electrode and reference electrode, while Zn NSAs/Al, S-ZnO NRAs/Al, or D-ZnO NRAs/Al were used as the working electrode, respectively. The diameter of the working electrode was 10 mm. The cell was first cycled at 0.05 mA between 0.01 V and 1.0 V for aging and activation. It was then discharged at different current densities and time, and charged to 1.0 V. For cycling performance measurements, Na was pre-plated on Zn NSAs/Al, S-ZnO NRAs/Al, and D-ZnO NRAs/Al with different areal capacities to obtain the Zn NSAs/Al-Na, S-ZnO NRAs/Al-Na, and D-ZnO NRAs/Al-Na anodes with different depth of discharge (DOD) values. For anode-less and anode-free cells, commercial $Na_3V_2O_2(PO_4)_2F$ (NVOPF) was used as a cathode. For the preparation of the cathode, NVOPF powder, Ketjen black, and polyvinylidene fluoride were mixed with a weight ratio of 8:1:1 with N-methyl-2-pyrrolidone as solvent. The slurry was then pasted on an Al foil and dried overnight at 150 °C in a vacuum oven. The electrode was cut into a plate with a diameter of 10 mm and the mass loading of active materials was about 1.6-1.8 mg cm^{-2}. The anode-less cell was assembled using NVOPF as the cathode and Zn NSAs/Al-Na, S-ZnO NRAs/Al-Na, or D-ZnO NRAs/Al-Na as the anode. The anode-free cell was assembled using NVOPF as the cathode and D-ZnO NRAs/Al as the anodic current collector.

Computational details

The Vienna ab initio simulation package was utilized for density functional theory calculations. The interactions between ions and electrons were modeled using the projector augmented wave method. The generalized gradient approximation based on the Perdew-Burke-Ernzerhof scheme was employed for the description of electron exchange and correlation interactions. Plane-wave basis with wave function cut-off energy of 450 eV was utilized. The atomic relaxation was terminated once the total energy tolerance converged to 10^{-5} eV and the changes of the force on atoms were less than 0.02 eV $Å^{-1}$. Additionally, all structures were optimized using a (3×3×1) Monkhorst-Pack K-point grid considering the symmetry of supercell, accuracy of calculation, and cost of used time. A vacuum space of 15 Å was placed along the Z axis to avoid the interaction among the slab.

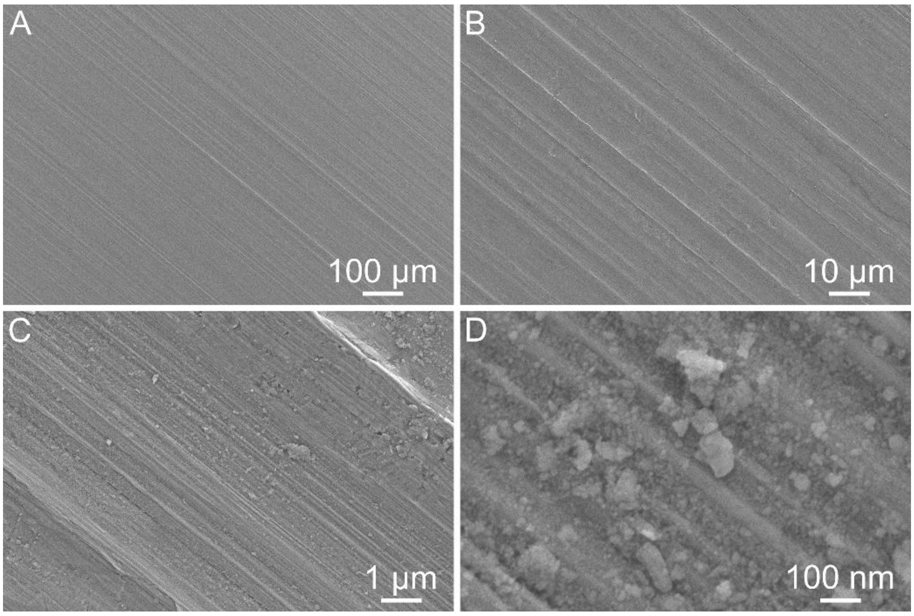

Figure S1. FESEM images of Al foil.

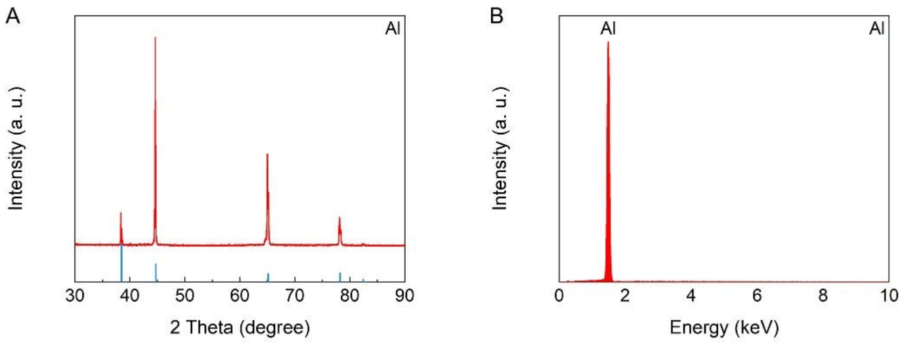

Figure S2. (A) XRD pattern and (B) EDX spectrum of Al foil.

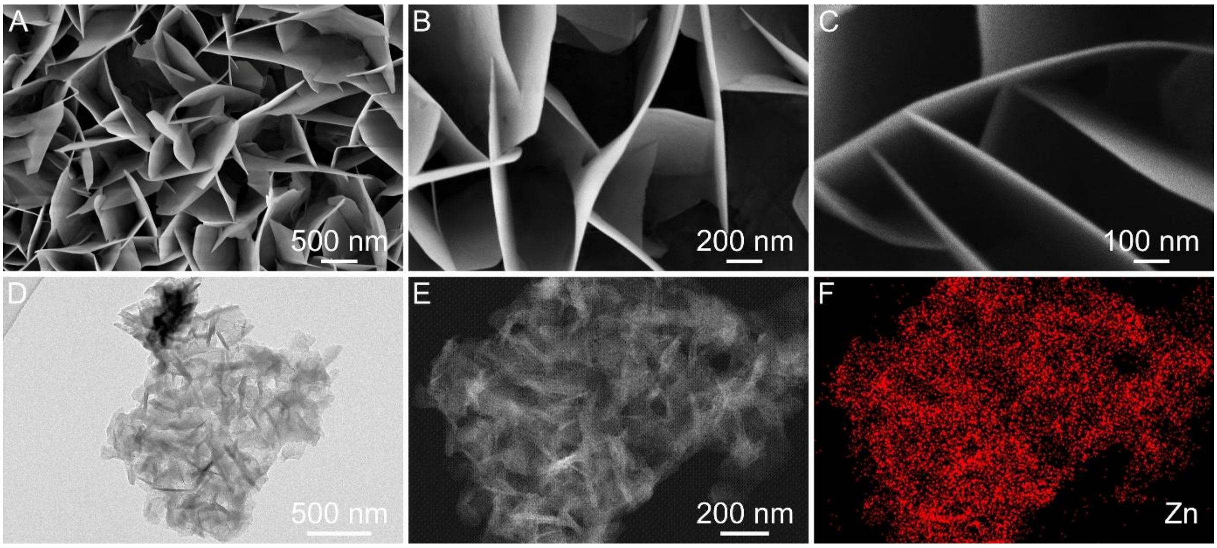

Figure S3. (A-C) FESEM, (D) TEM, (E, F) HAADF-STEM and elemental mapping images of Zn NSAs/Al.

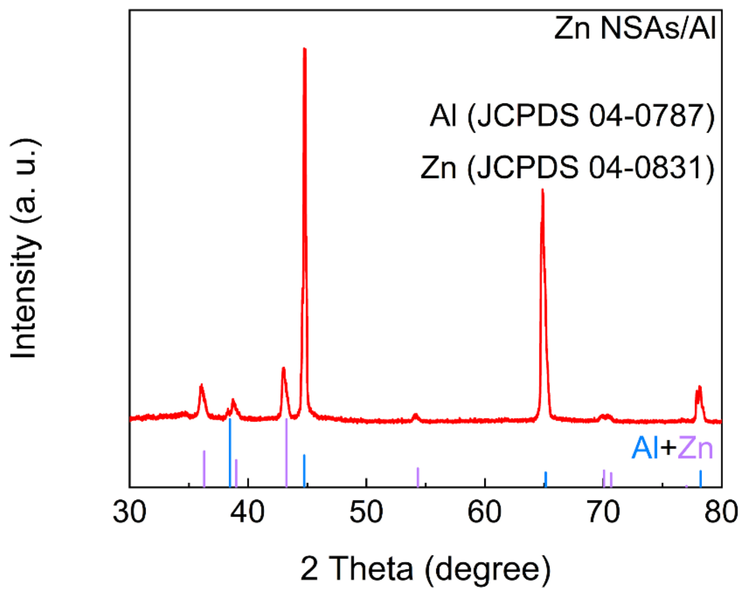

Figure S4. XRD pattern of Zn NSAs/Al.

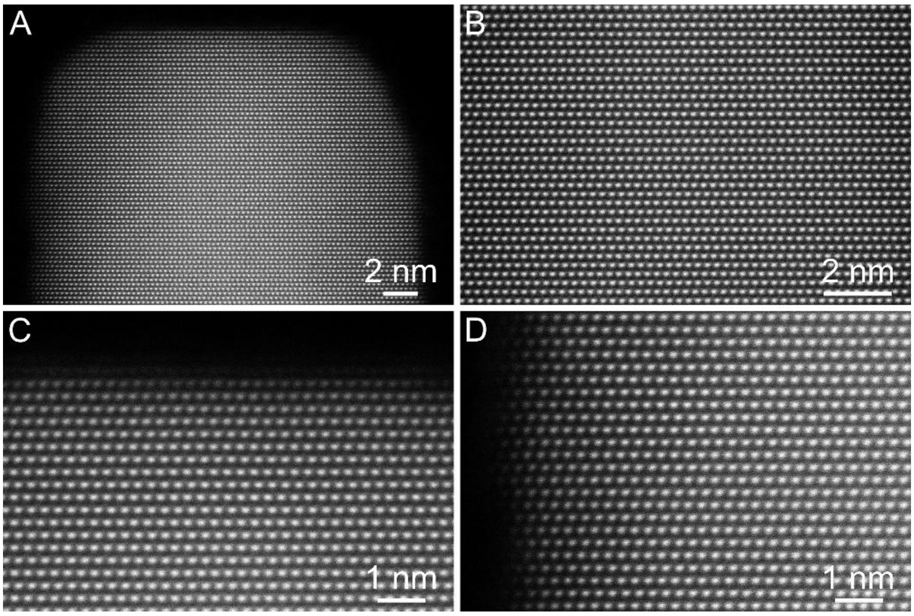

Figure S5. HAADF-STEM images of D-ZnO NRAs/Al.

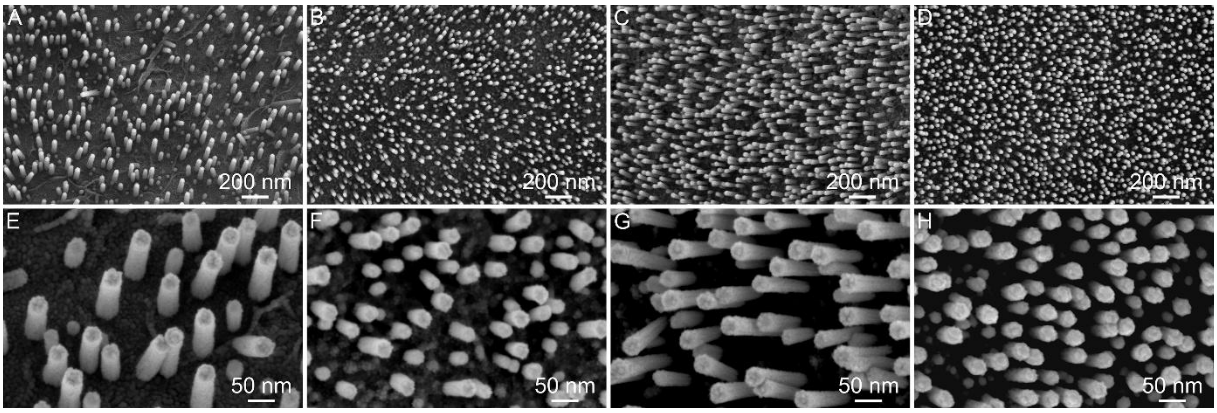

Figure S6. FESEM images of ZnO NRAs/Al synthesized at different temperatures. (A, E) 440 ℃, (B, F) 460 ℃, (C, G) 480 ℃, and (D, H) 500 ℃.

The ZnO NRAs/Al synthesized at 440 ℃ and 500 ℃ are denoted as S-ZnO NRAs/Al and D-ZnO NRAs/Al, respectively.

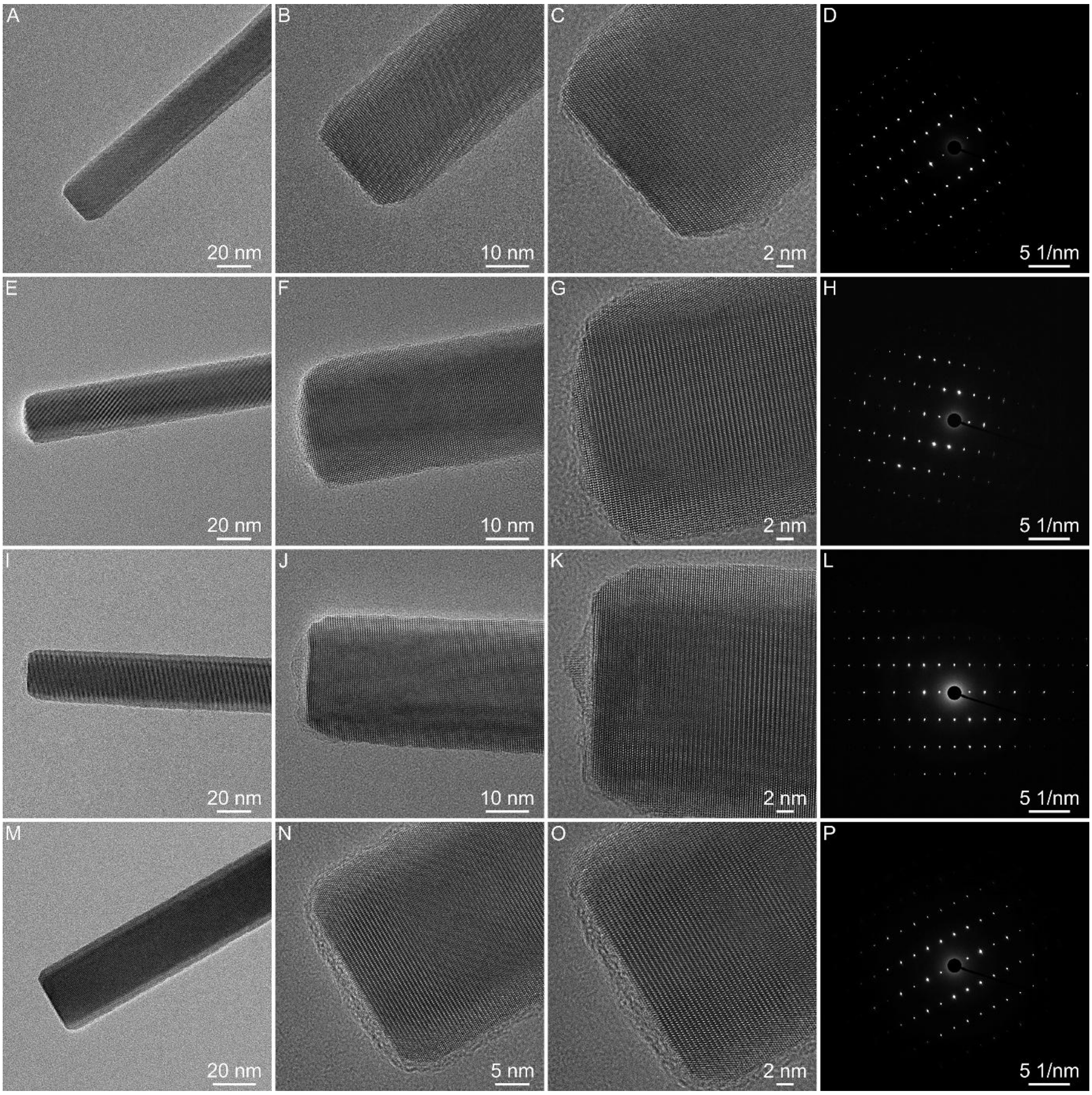

Figure S7. (A-C, E-G, I-K, M-O) HRTEM and (D, H, L, P) electronic diffraction images of ZnO NRAs/Al synthesized at different temperatures. (A-D) 440 ℃, (E-H) 460 ℃, (I-L) 480 ℃, and (M-P) 500 ℃.

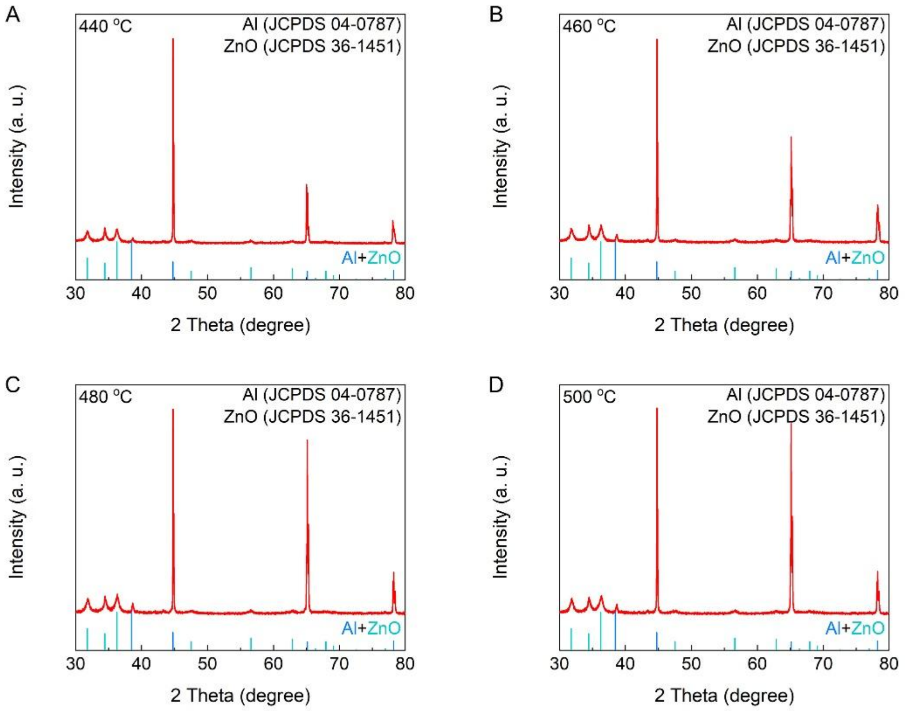

Figure S8. XRD patterns of ZnO NRAs/Al synthesized at different temperatures. (A) 440 ℃, (B) 460 ℃, (C) 480 ℃, and (D) 500 ℃.

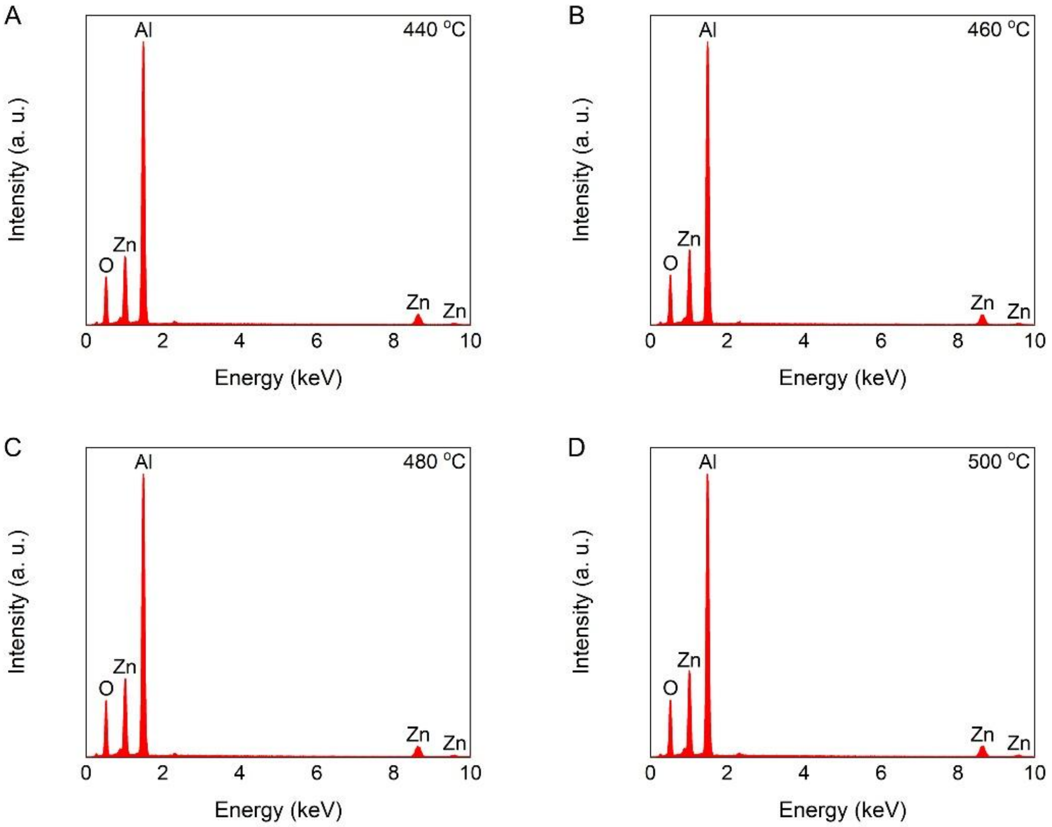

Figure S9. EDX spectra of ZnO NRAs/Al synthesized at different temperatures. (A) 440 °C, (B) 460 °C, (C) 480 °C, and (D) 500 °C.

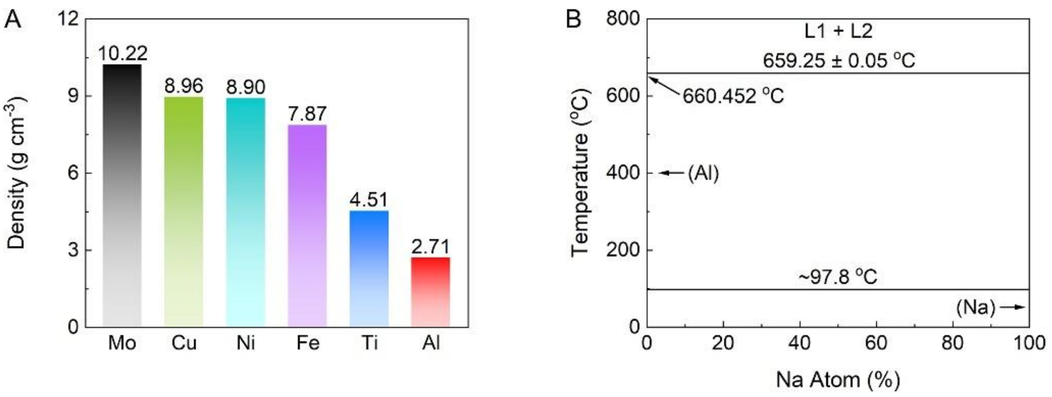

Figure S10. Density and phase diagram analyses. (A) Density of several current collectors. (B) Phase diagram of Na-Al.

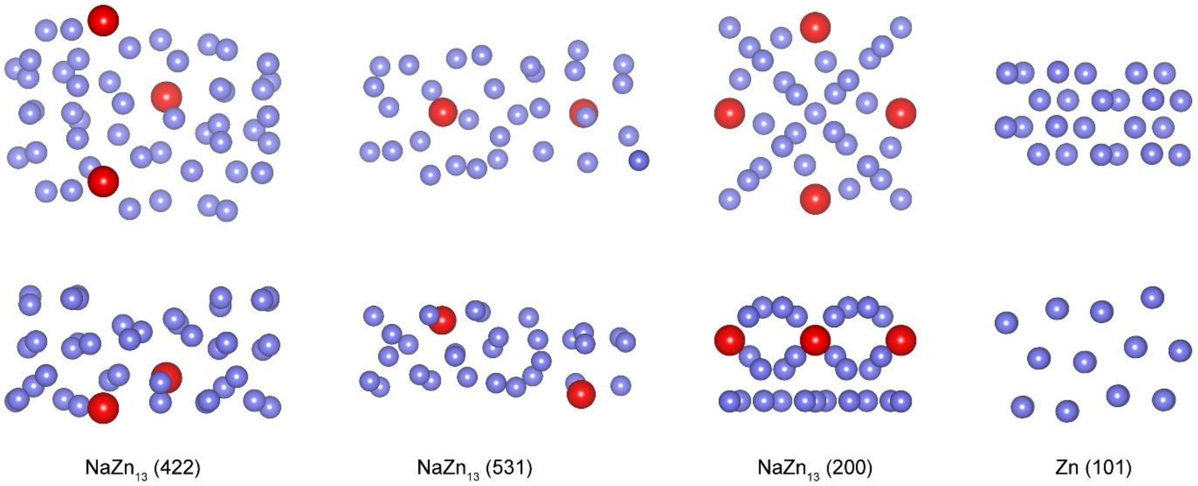

Figure S11. Models of $NaZn_{13}$ (422), $NaZn_{13}$ (531), $NaZn_{13}$ (200), and Zn (101).

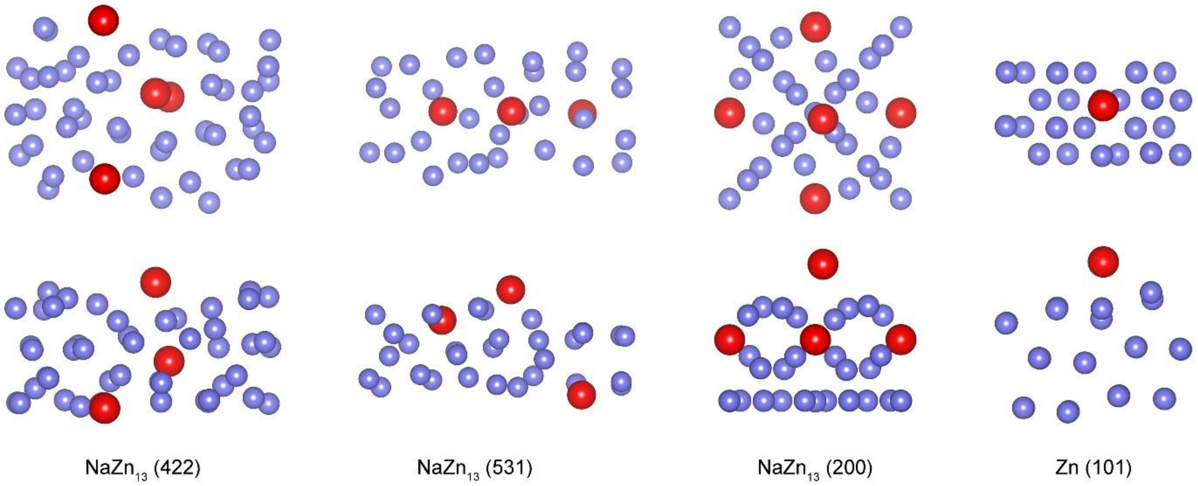

Figure S12. Models of $NaZn_{13}$ (422), $NaZn_{13}$ (531), $NaZn_{13}$ (200), and Zn (101) after Na atom adsorption.

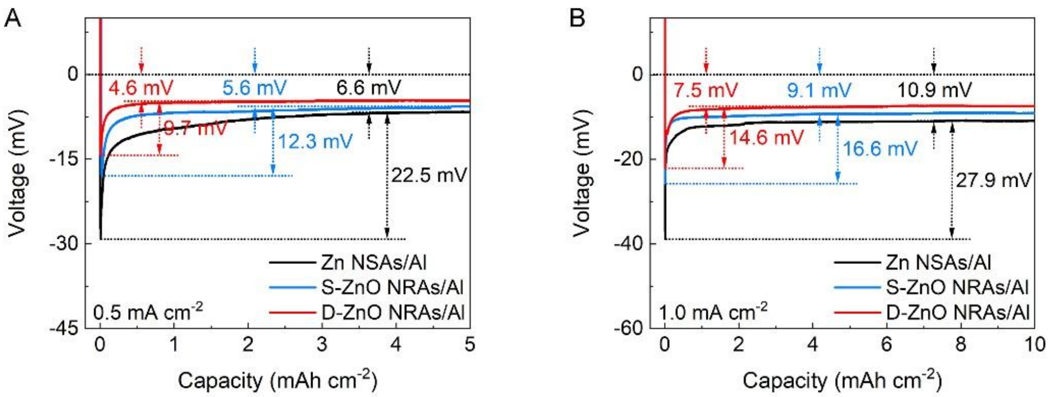

Figure S13. Voltage-capacity curves during Na nucleation on different hosts tested at different current densities. (A) 0.5 mA cm^{-2} and (B) 1.0 mA cm^{-2}.

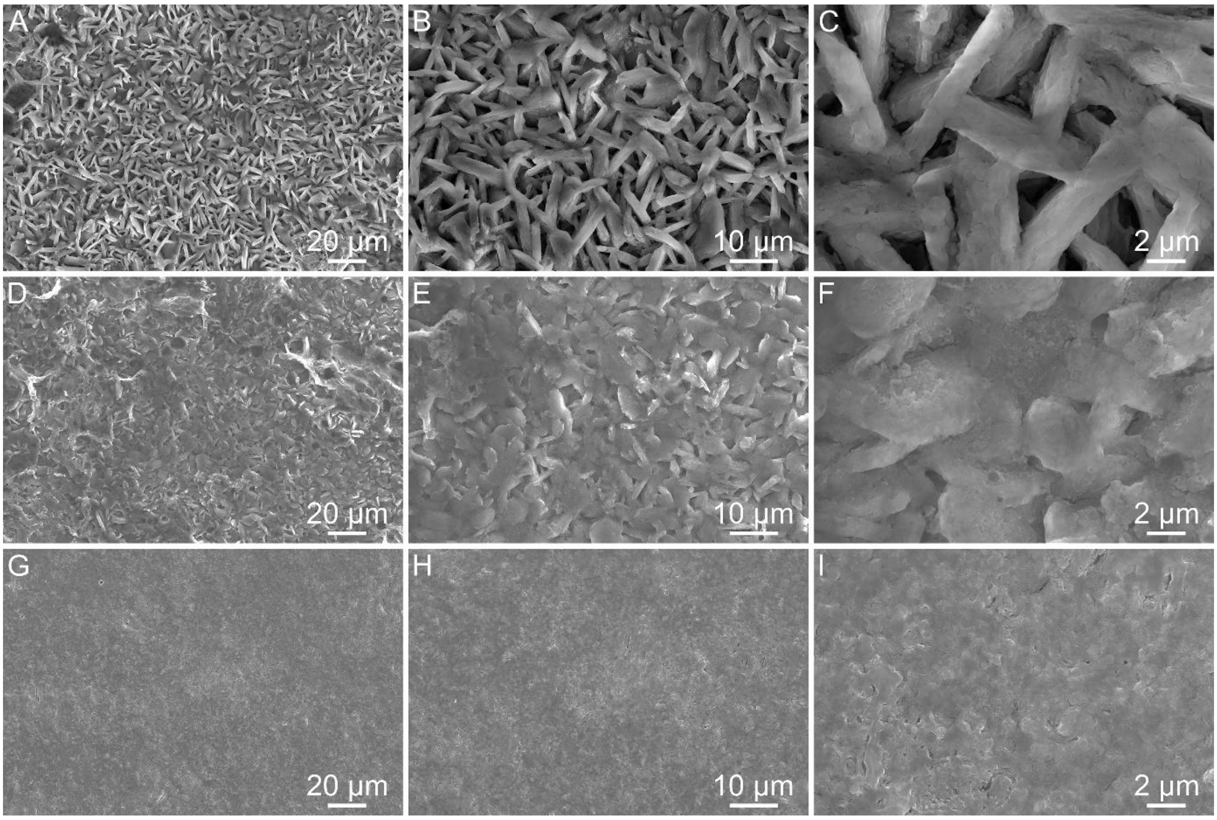

Figure S14. FESEM images of different hosts after Na deposition at an areal capacity of 20 mAh cm^{-2}. (A-C) Zn NSAs/Al, (D-F) S-ZnO NRAs/Al, and (G-I) D-ZnO NRAs/Al.

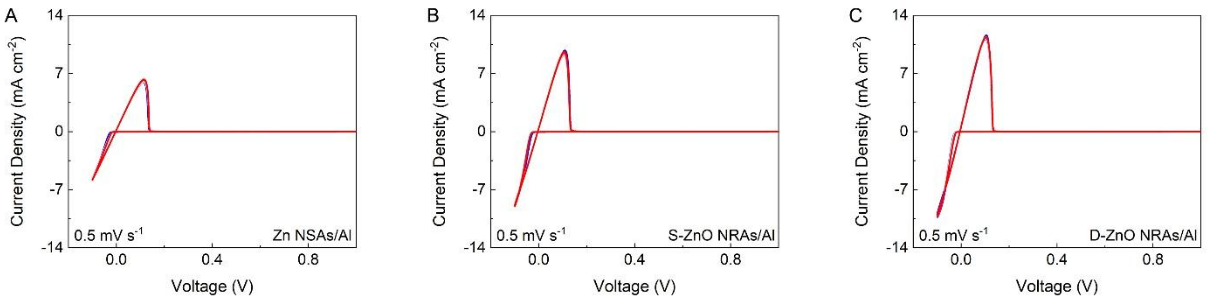

Figure S15. CV curves of different hosts tested at a scan rate of 0.5 mV s^{-1}. (A) Zn NSAs/Al, (B) S-ZnO NRAs/Al, and (C) D-ZnO NRAs/Al.

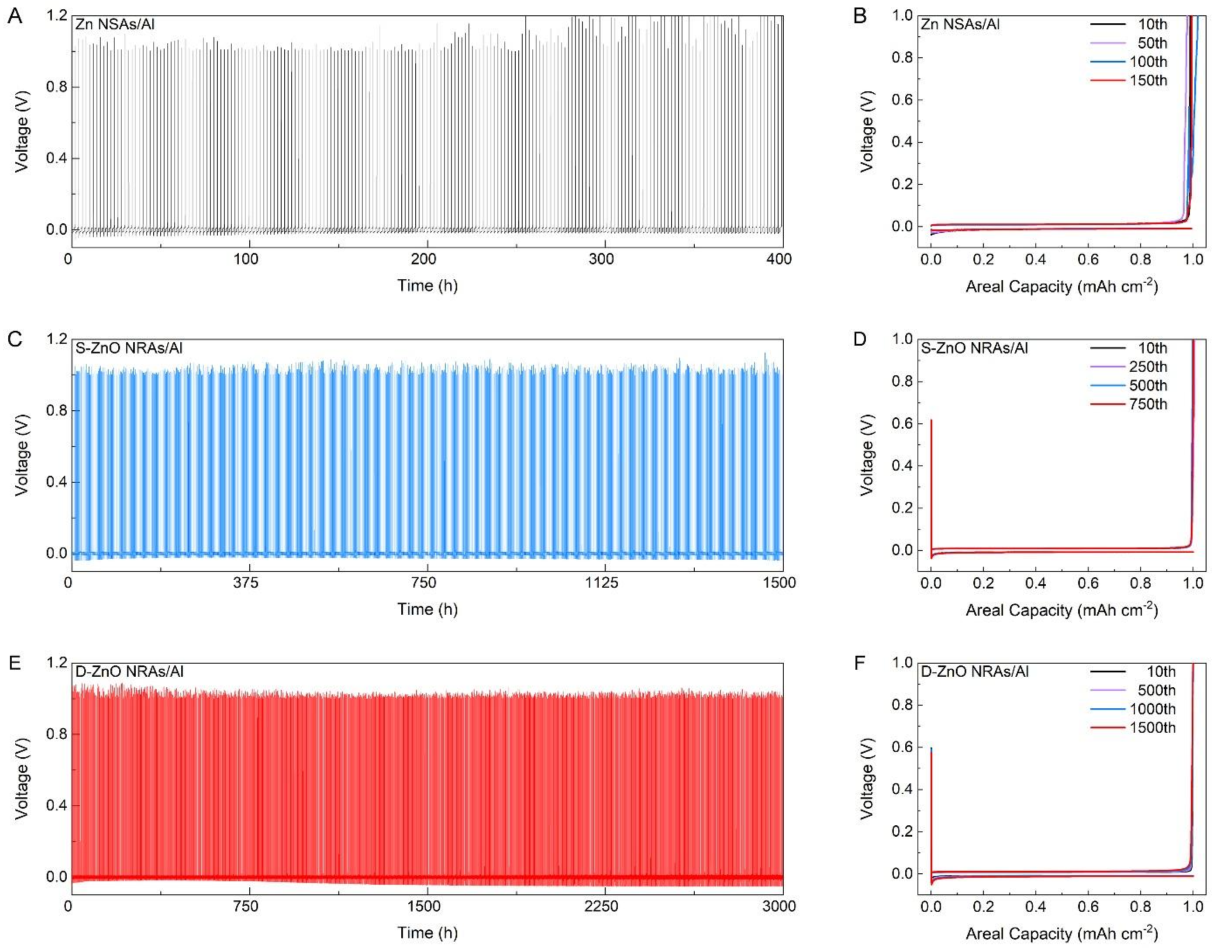

Figure S16. (A, C, E) Voltage-time and (B, D, F) voltage-capacity curves of different hosts tested 1 mA cm^{-2} and 1 mAh cm^{-2}. (A, B) Zn NSAs/Al, (C, D) S-ZnO NRAs/Al, and (E, F) D-ZnO NRAs/Al.

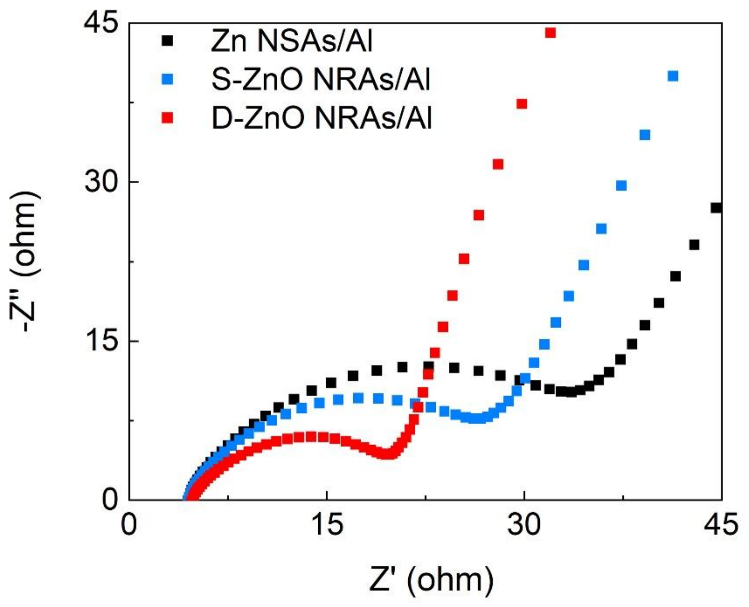

Figure S17. Nyquist plots of different hosts after cycling.

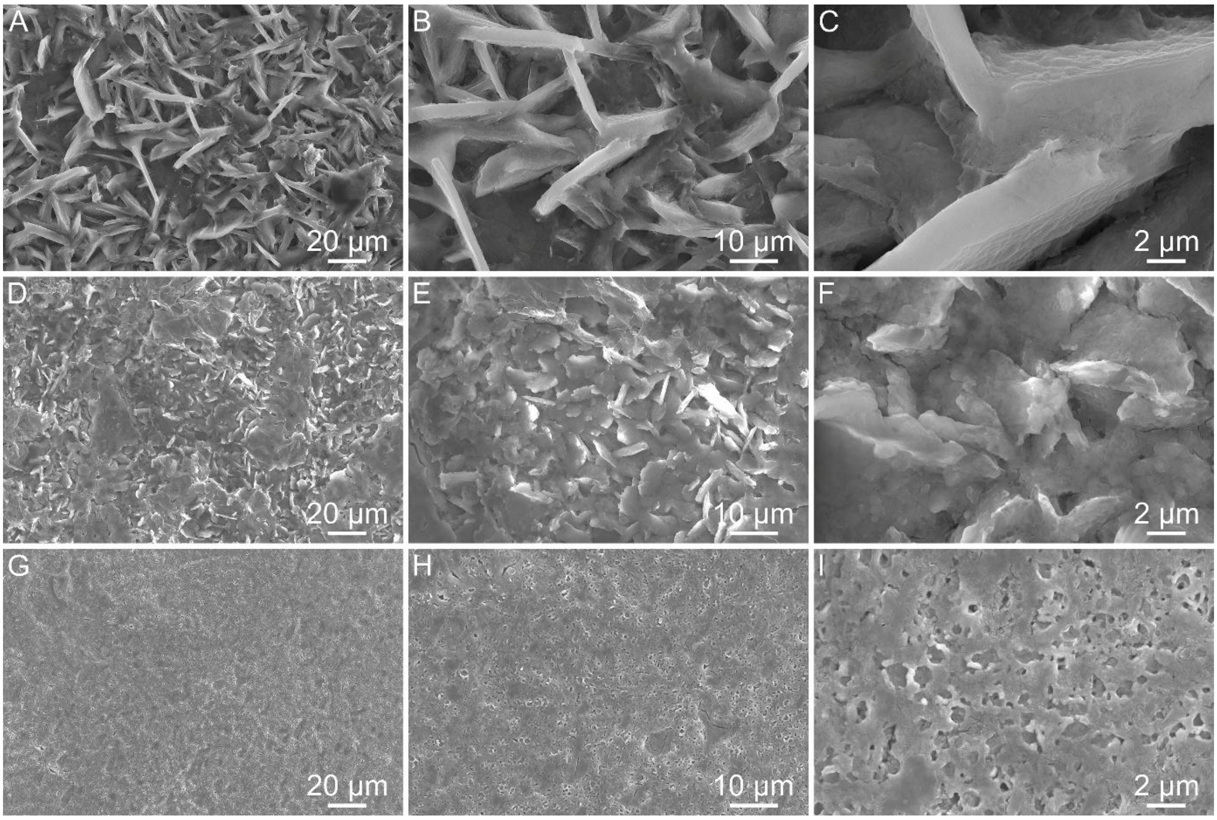

Figure S18. FESEM images of different hosts after cycling. (A-C) Zn NSAs/Al, (D-F) S-ZnO NRAs/Al, and (G-I) D-ZnO NRAs/Al.

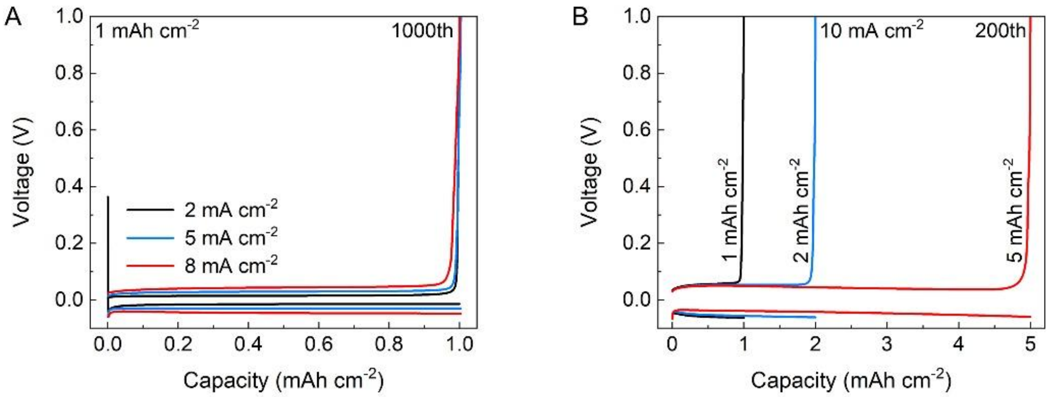

Figure S19. Voltage-capacity curves of D-ZnO NRAs/Al host tested at different (A) current densities and (B) areal capacities.

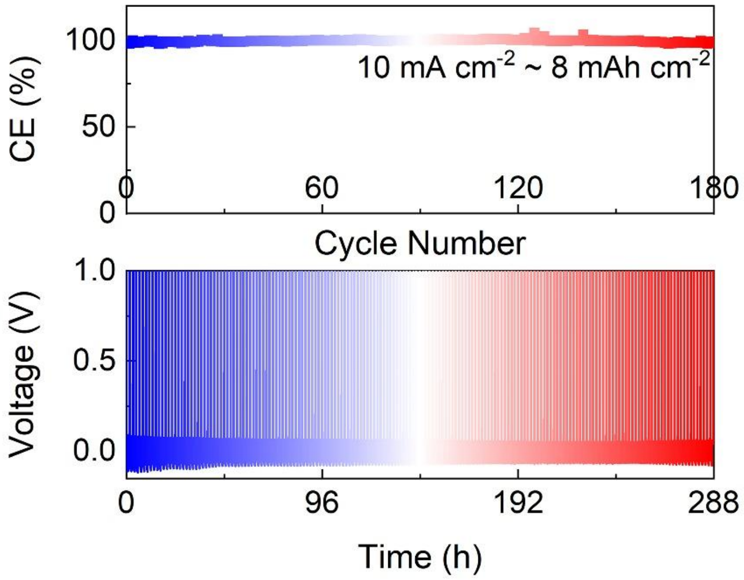

Figure S20. CE plot and voltage-time curves of D-ZnO NRAs/Al host tested at 10 mA cm^{-2} and 8 mAh cm^{-2}.

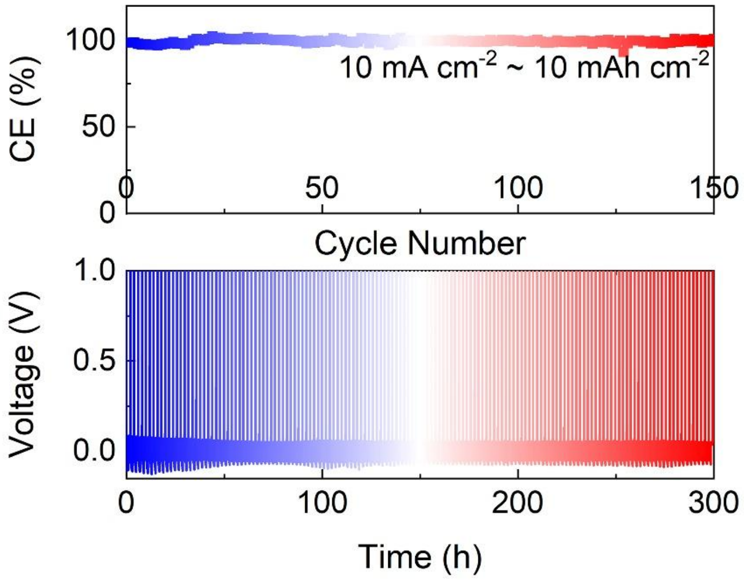

Figure S21. CE plot and voltage-time curves of D-ZnO NRAs/Al host tested at 10 mA cm^{-2} and 10 mAh cm^{-2}.

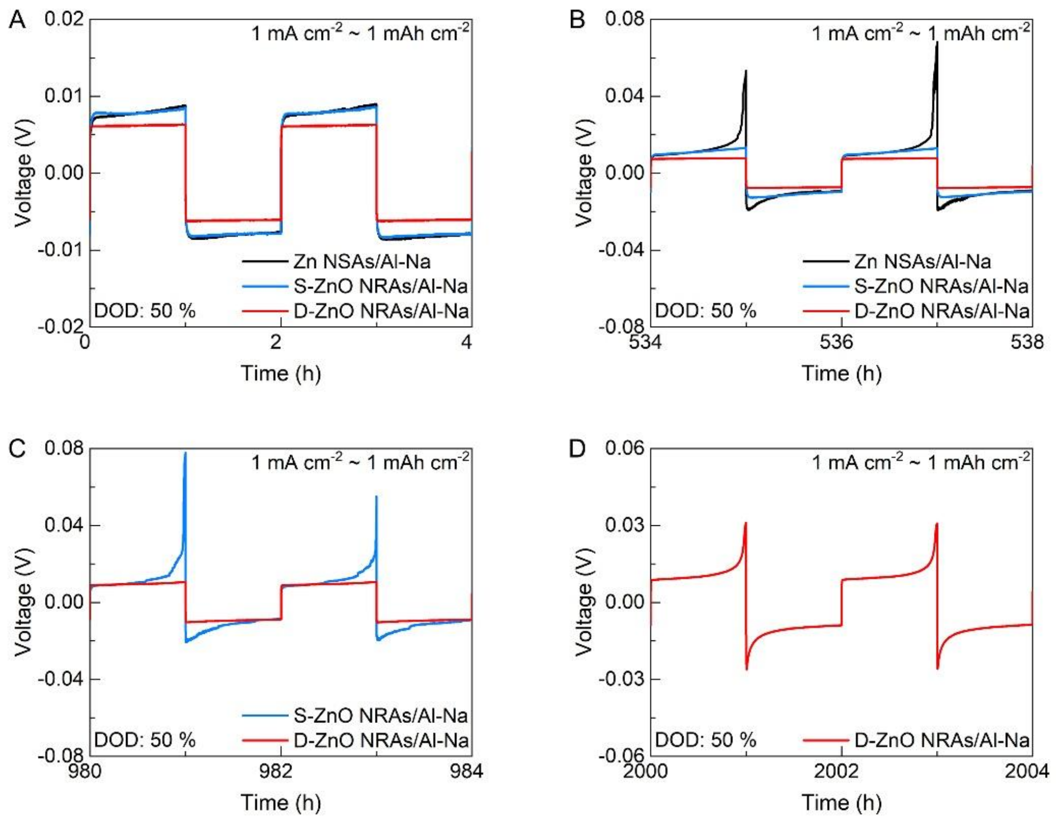

Figure S22. Voltage-time curves of different electrodes tested at 1 mA cm^{-2} and 1 mAh cm^{-2} with a DOD of 50 %.

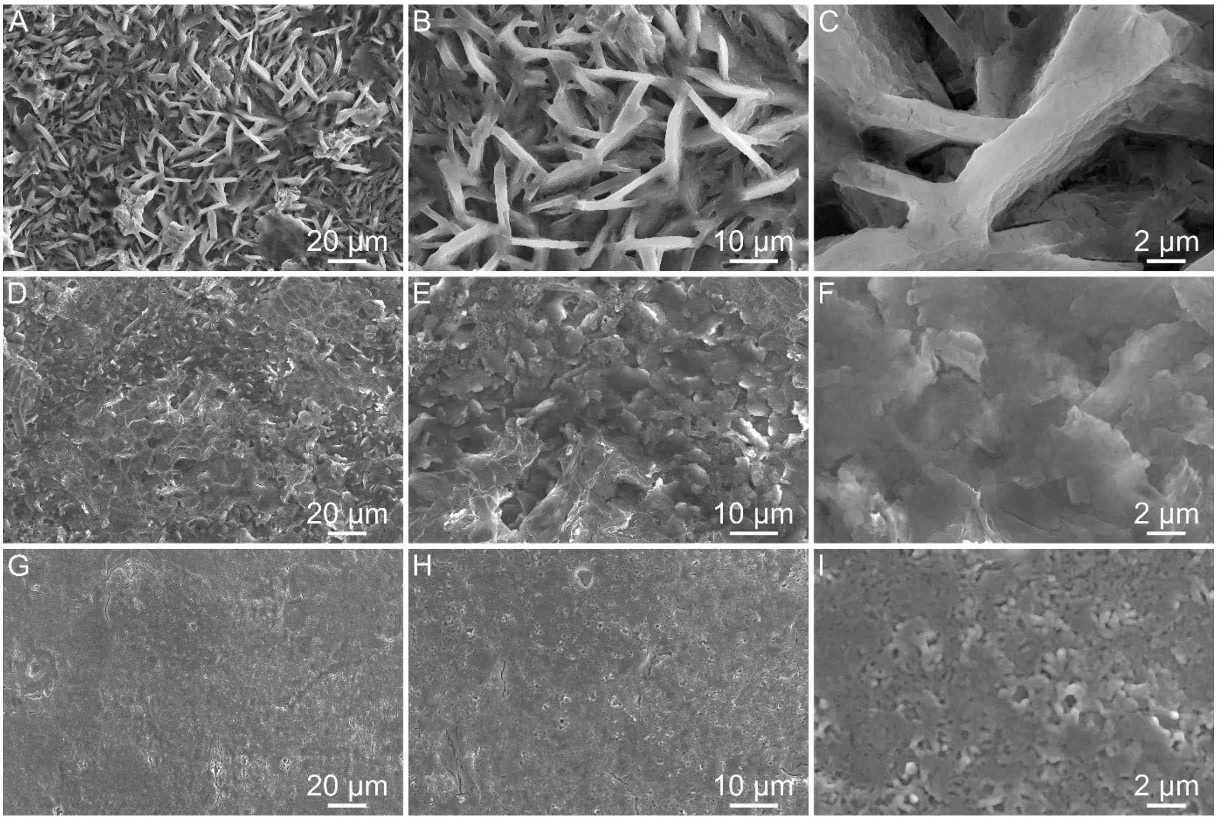

Figure S23. FESEM images of different electrodes after cycling. (A-C) Zn NSAs/Al-Na, (D-F) S-ZnO NRAs/Al-Na, and (G-I) D-ZnO NRAs/Al-Na.

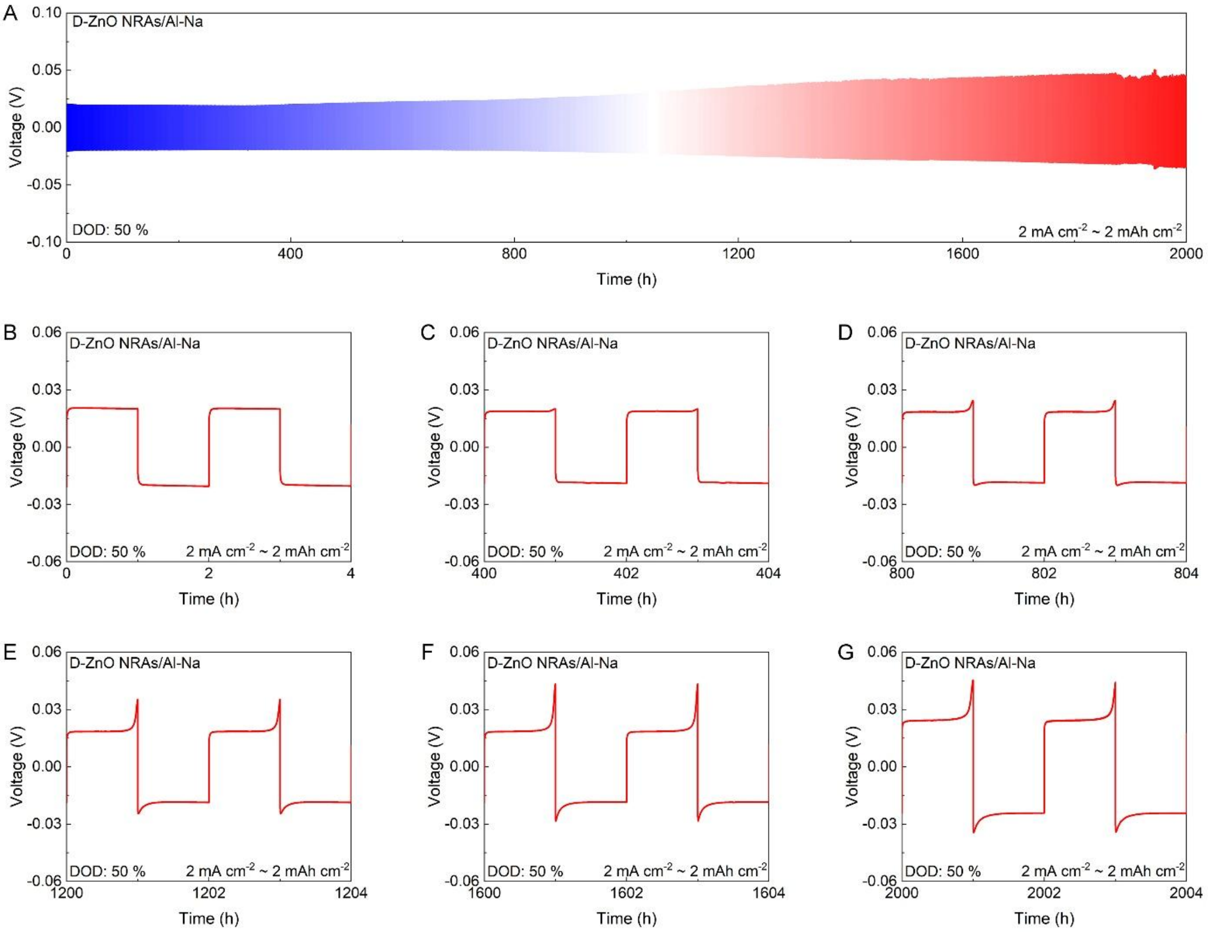

Figure S24. (A) Cycling performance and (B-G) voltage-time curves of D-ZnO NRAs/Al-Na electrode tested at 2 mA cm^{-2} and 2 mAh cm^{-2} with a DOD of 50 %.

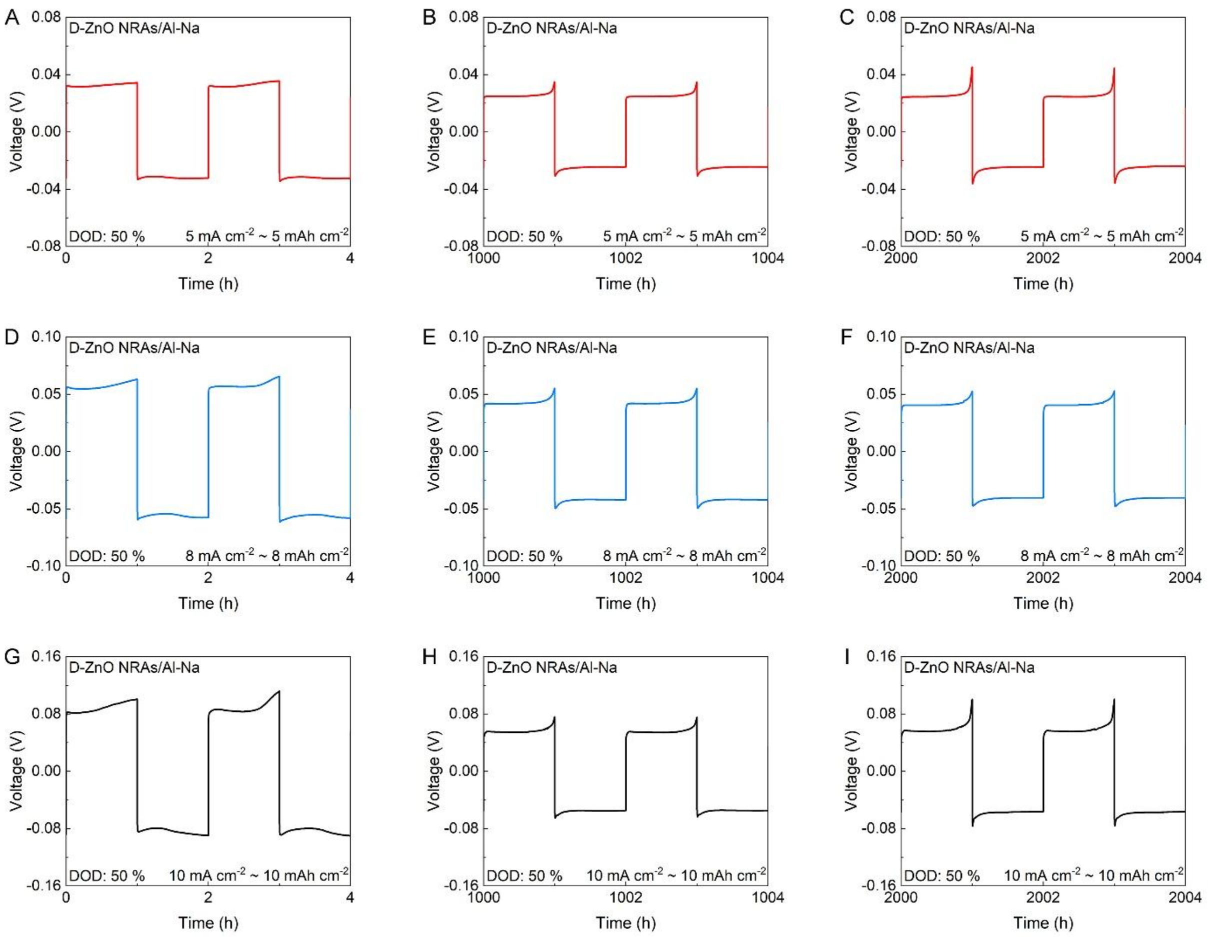

Figure S25. Voltage-time curves of D-ZnO NRAs/Al-Na electrode tested at different current densities and areal capacities. (A-C) 5 mA cm^{-2} and 5 mAh cm^{-2}, (D-F) 8 mA cm^{-2} and 8 mAh cm^{-2}, (G-I) 10 mA cm^{-2} and 10 mAh cm^{-2}.

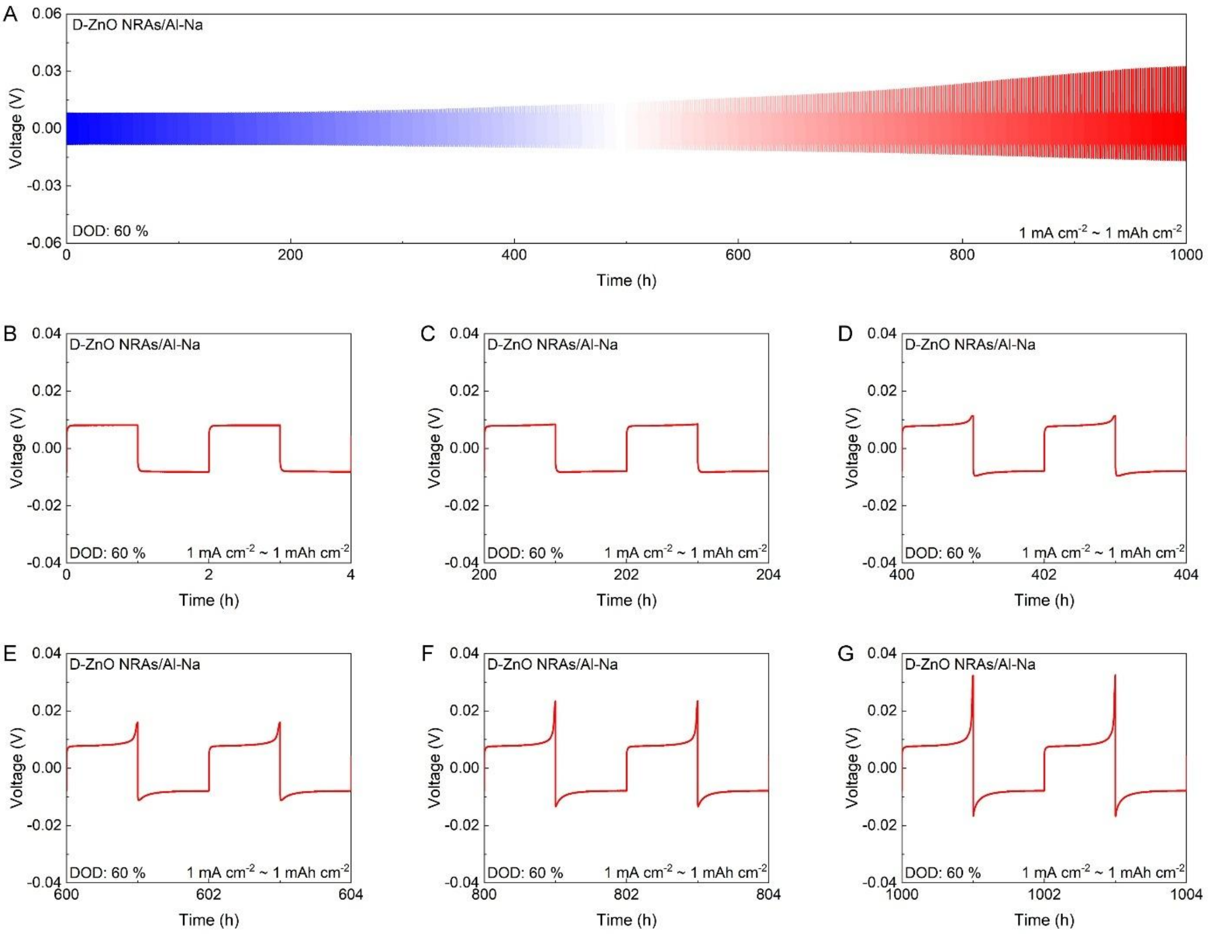

Figure S26. (A) Cycling performance and (B-G) voltage-time curves of D-ZnO NRAs/Al-Na electrode tested at 1 mA cm^{-2} and 1 mAh cm^{-2} with a DOD of 60 %.

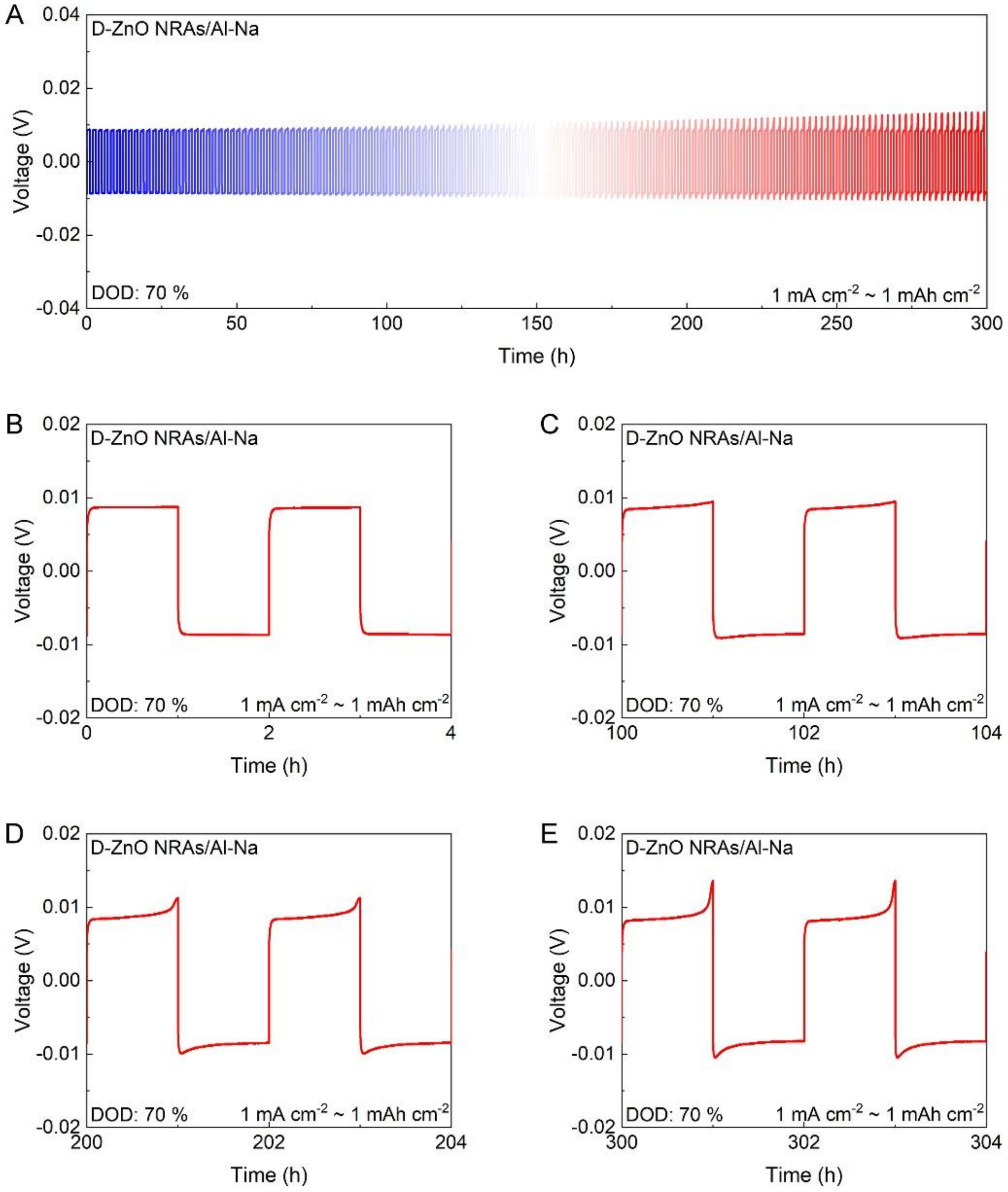

Figure S27. (A) Cycling performance and (B-E) voltage-time curves of D-ZnO NRAs/Al-Na electrode tested at 1 mA cm^{-2} and 1 mAh cm^{-2} with a DOD of 70 %.

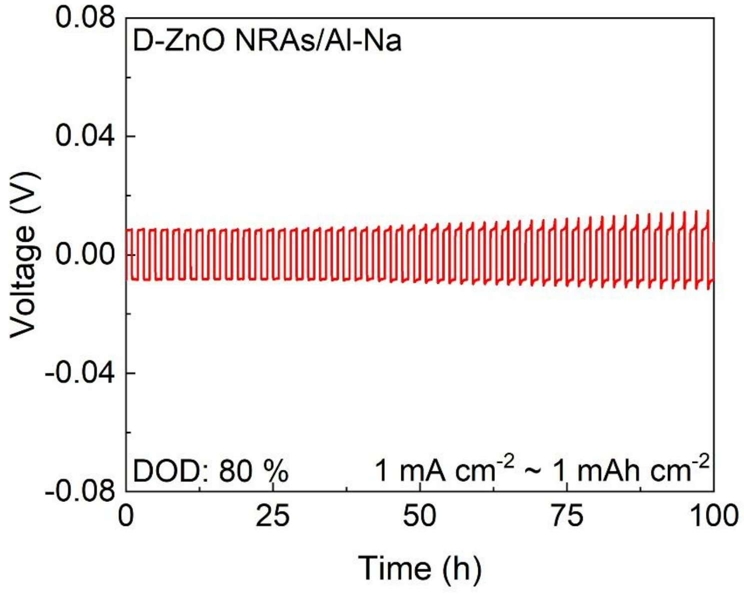

Figure S28. Cycling performance of D-ZnO NRAs/Al-Na electrode tested at 1 mA cm^{-2} and 1 mAh cm^{-2} with a DOD of 80 %.

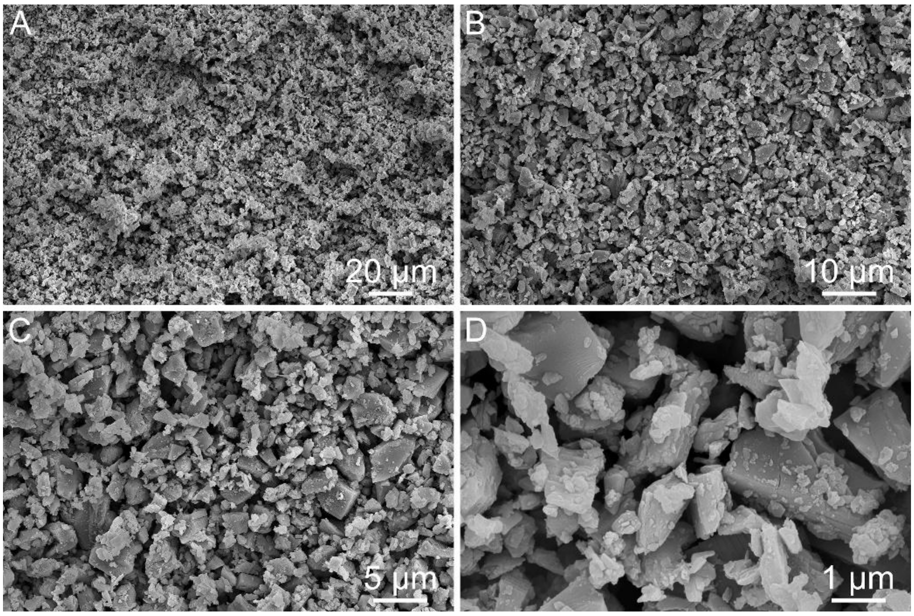

Figure S29. FESEM images of NVOPF.

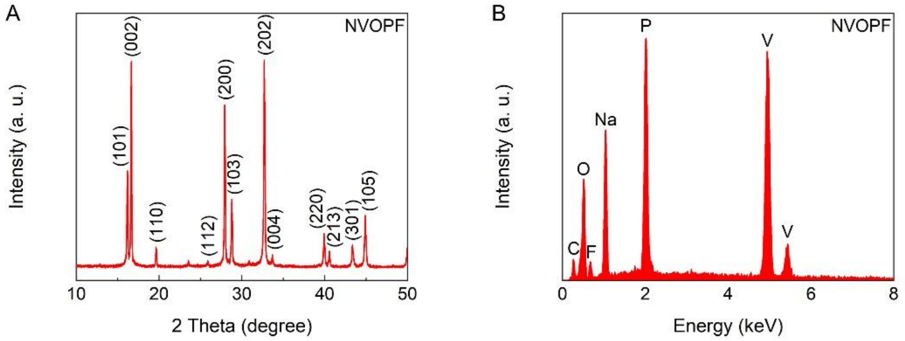

Figure S30. (A) XRD pattern and (B) EDX spectrum of NVOPF.

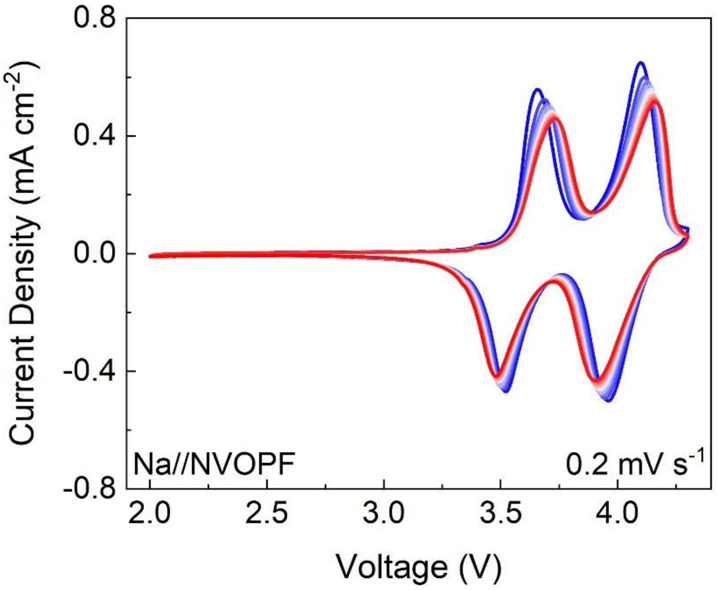

Figure S31. CV curve of Na//NVOPF cell tested at 0.2 mV s^{-1}.

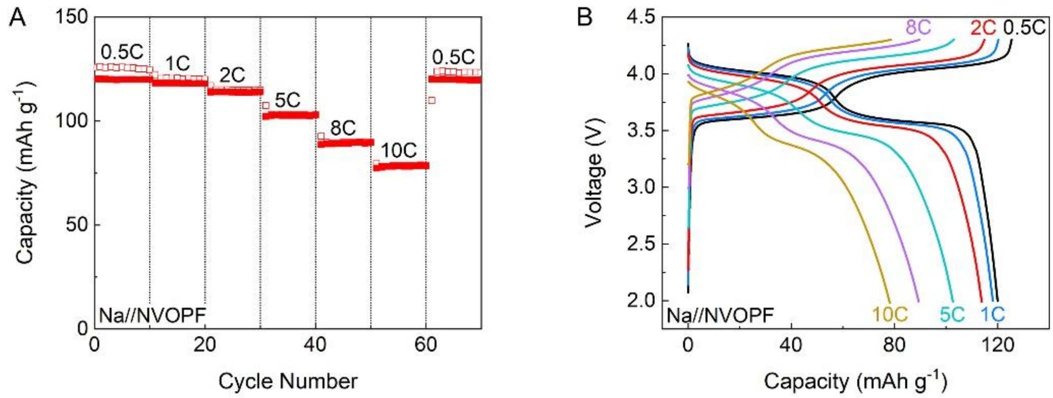

Figure S32. Rate performance of Na//NVOPF cell tested at different current densities.

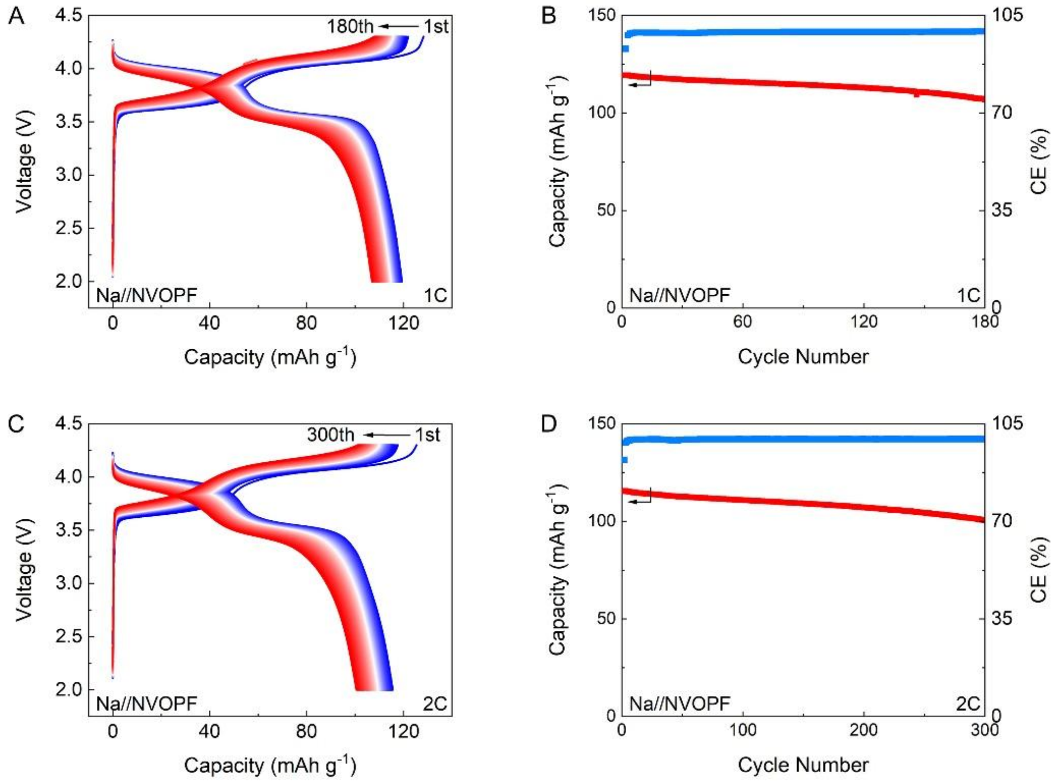

Figure S33. (A, C) Charge-discharge voltage curves and (B, D) cycling performance of Na//NVOPF cell tested at (A, B) 1C and (C, D) 2C.

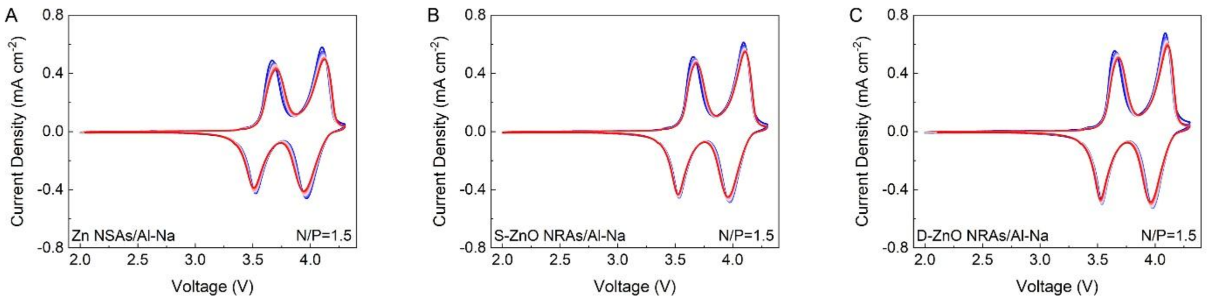

Figure S34. CV curves of different full cells with an N/P ratio of 1.5 tested at a scan rate of 0.2 mV s^{-1}. (A) Zn NSAs/Al-Na//NVOPF, (B) S-ZnO NRAs/Al-Na//NVOPF, and (C) D-ZnO NRAs/Al-Na//NVOPF.

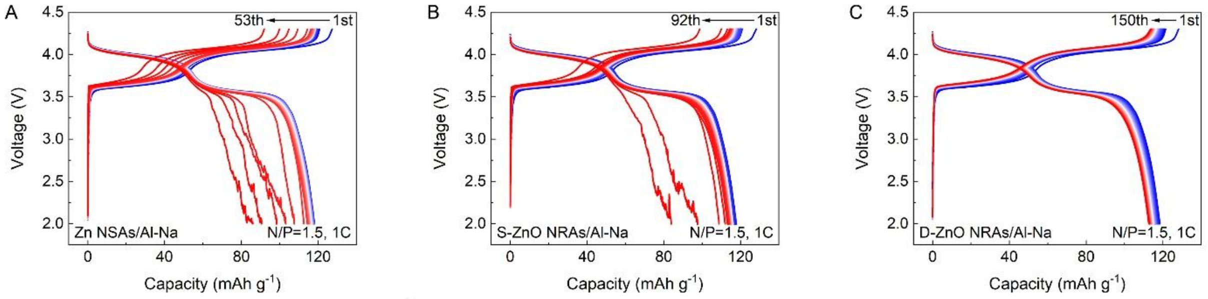

Figure S35. Charge-discharge voltage curves of different full cells with an N/P ratio of 1.5 tested at 1C. (A) Zn NSAs/Al-Na//NVOPF, (B) S-ZnO NRAs/Al-Na//NVOPF, and (C) D-ZnO NRAs/Al-Na//NVOPF.

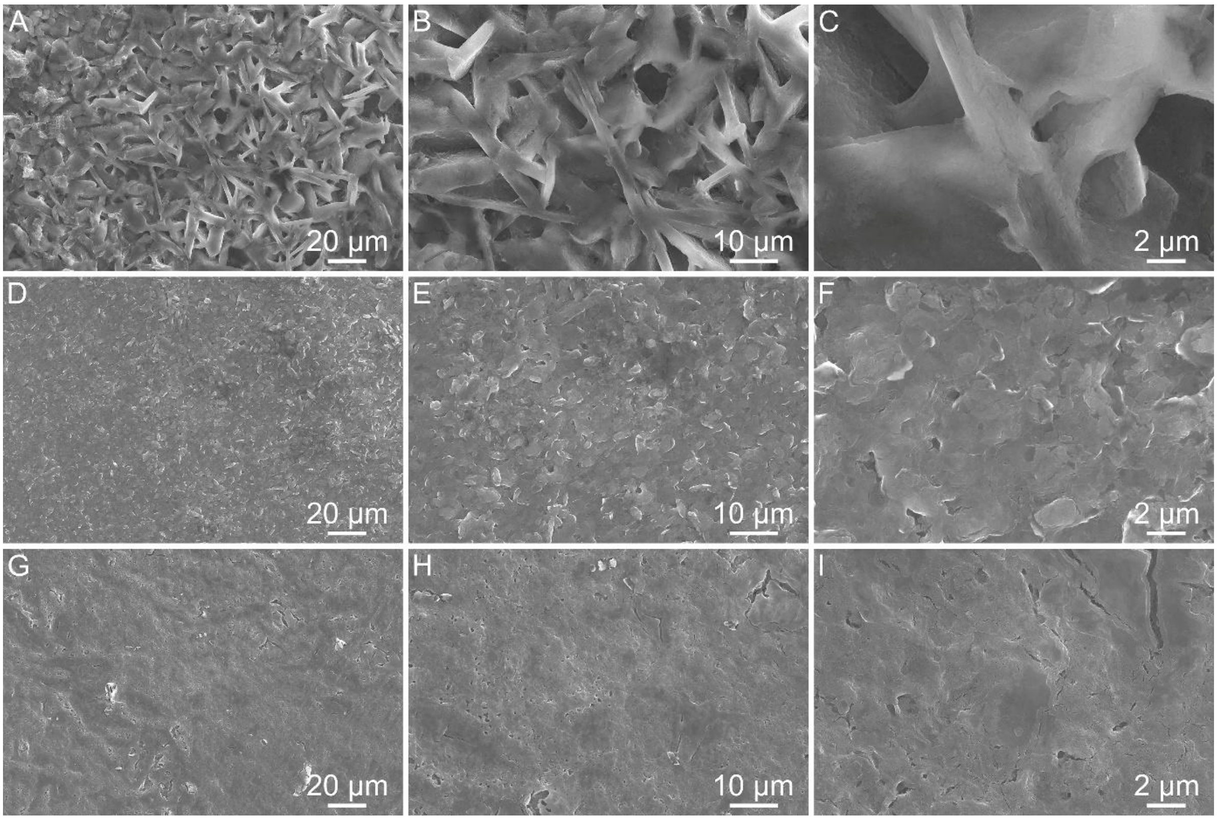

Figure S36. FESEM images of different anodes in full cell after cycling. (A-C) Zn NSAs/Al-Na, (D-F) S-ZnO NRAs/Al-Na, and (G-I) D-ZnO NRAs/Al-Na.

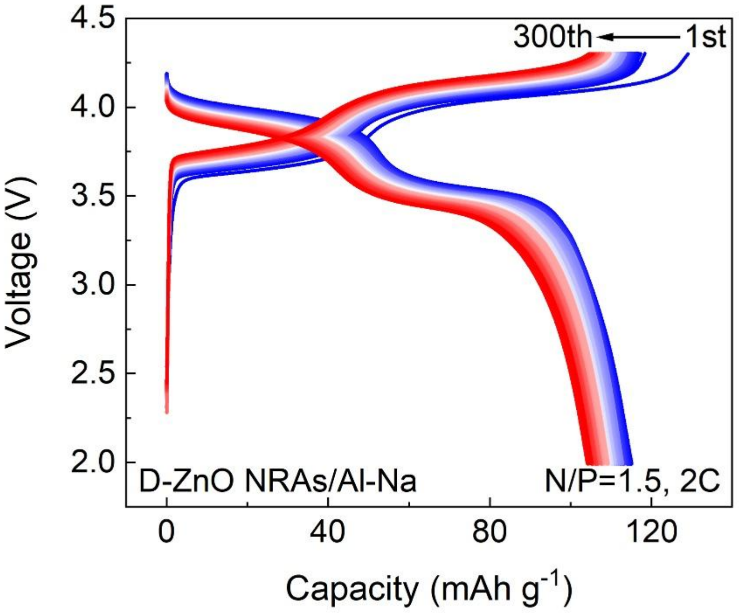

Figure S37. Charge-discharge voltage curves of D-ZnO NRAs/Al-Na//NVOPF cell with an N/P ratio of 1.5 tested at 2C.

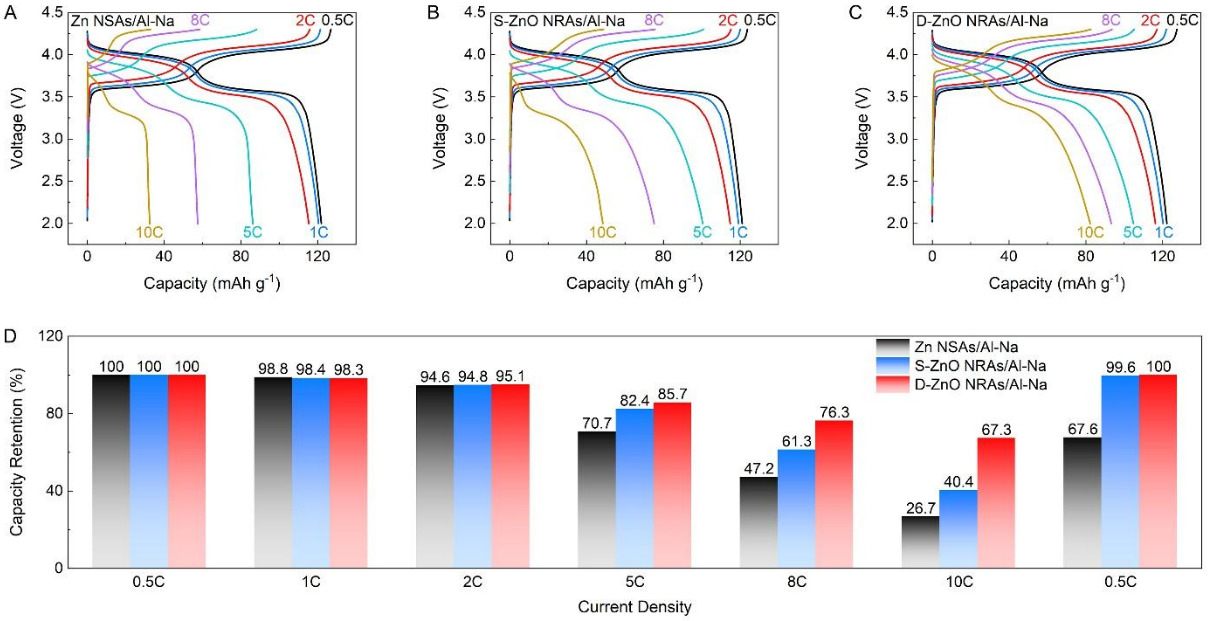

Figure S38. (A-C) Charge-discharge voltage curves and (D) capacity retention of different full cells with an N/P ratio of 1.5 tested at different current densities. (A) Zn NSAs/Al-Na//NVOPF, (B) S-ZnO NRAs/Al-Na//NVOPF, and (C) D-ZnO NRAs/Al-Na//NVOPF.

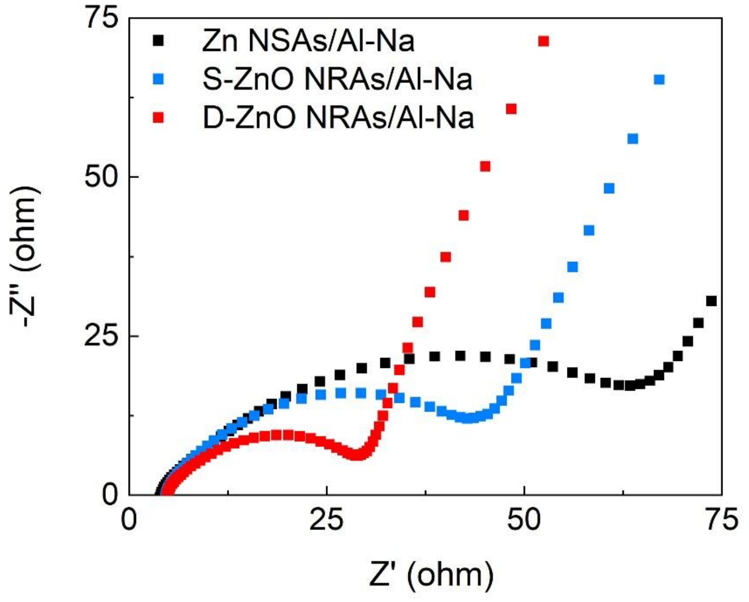

Figure S39. Nyquist plots of full cells after cycling.

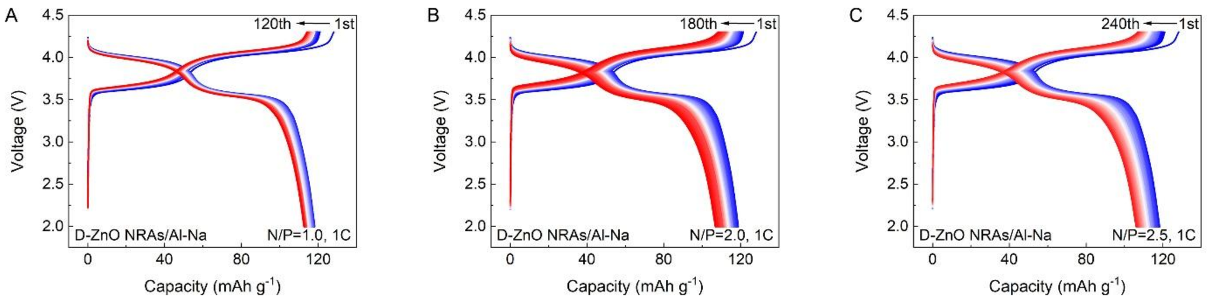

Figure S40. Charge-discharge voltage curves of D-ZnO NRAs/Al-Na//NVOPF cell with different N/P ratios tested at 1C. (A) 1.0, (B) 2.0, and (C) 2.5.

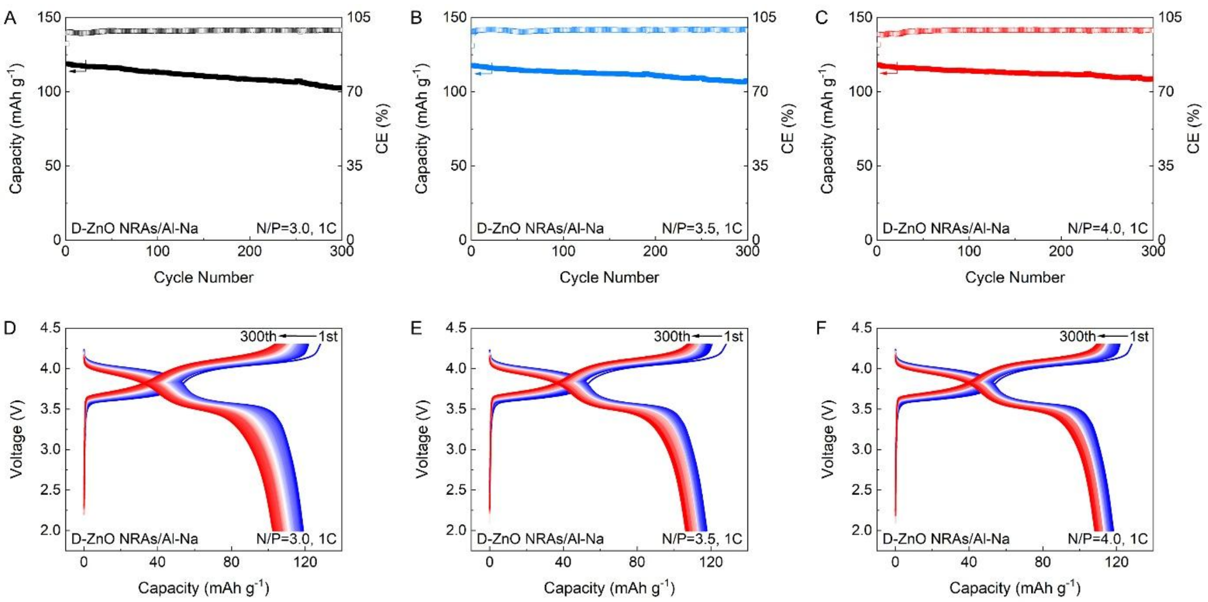

Figure S41. (A-C) Cycling performance and (D-F) charge-discharge voltage curves of D-ZnO NRAs/Al-Na//NVOPF cell with different N/P ratios tested at 1C. (A, D) 3.0, (B, E) 3.5, and (C, F) 4.0.

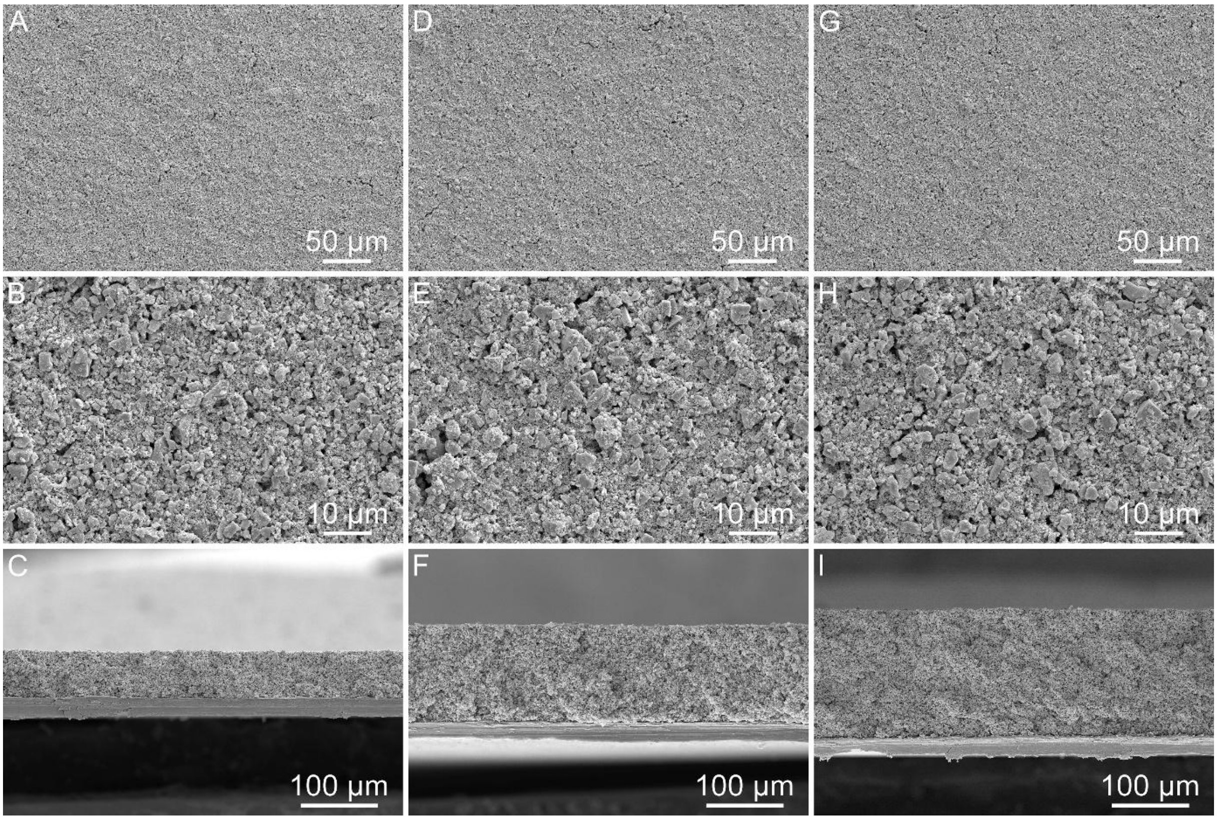

Figure S42. (A, B, D, E, G, H) Top-view and (C, F, I) cross-sectional FESEM images of NVOPF cathode with different mass loadings of (A-C) 9.43 mg cm^{-2}, (D-F) 22.44 mg cm^{-2}, and (G-I) 32.62 mg cm^{-2}.

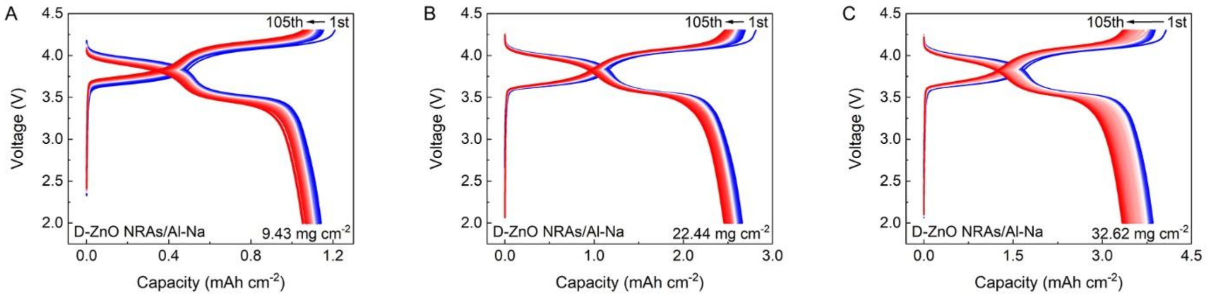

Figure S43. Charge-discharge voltage curves of D-ZnO NRAs/Al-Na//NVOPF cell with different NVOPF loadings. (A) 9.43 mg cm^{-2}, (B) 22.44 mg cm^{-2}, and (C) 32.62 mg cm^{-2}.

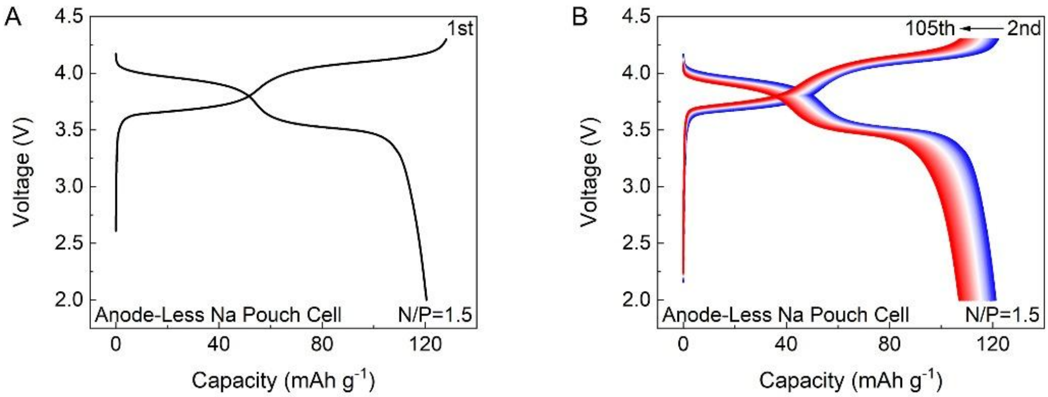

Figure S44. (A) 1st and (B) 2nd to 105th charge-discharge voltage curves of D-ZnO NRAs/Al-Na//NVOPF pouch cell with an N/P ratio of 1.5.

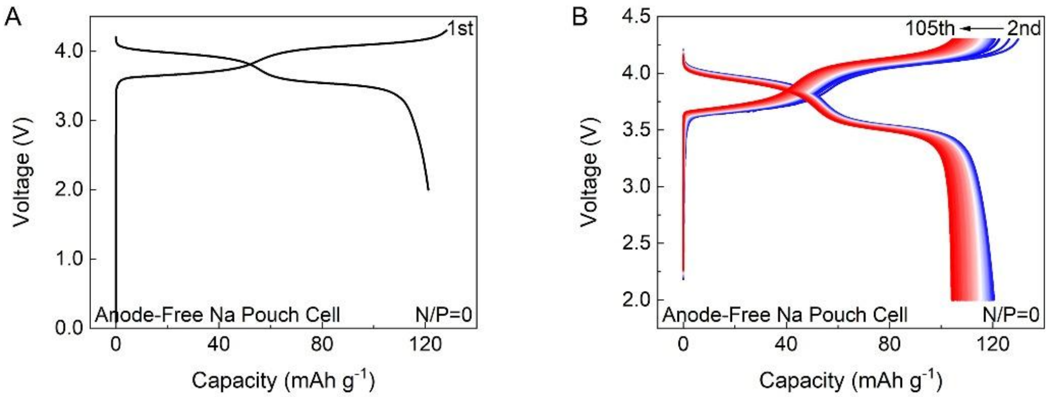

Figure S45. (A) 1st and (B) 2nd to 105th charge-discharge voltage curves of D-ZnO NRAs/Al//NVOPF pouch cell with an N/P ratio of 0.

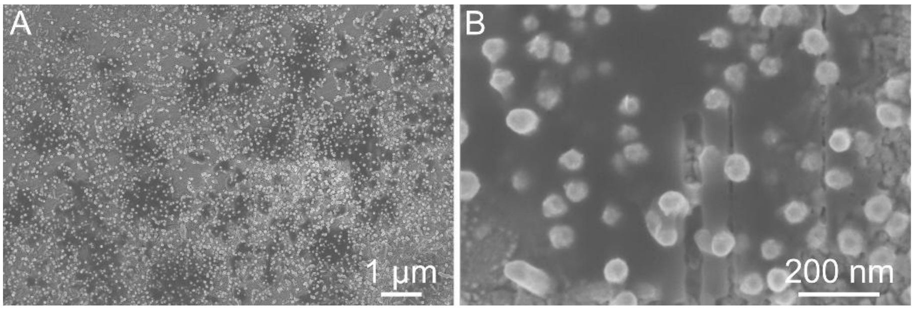

Figure S46. FESEM images of D-ZnO NRAs/Al in anode-free pouch cell after cycling.

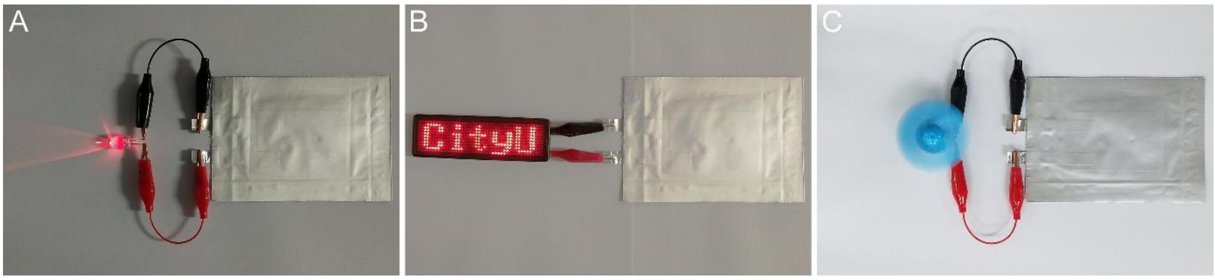

Figure S47. Optical images of fully charged D-ZnO NRAs/Al//NVOPF pouch cell to light up (A) an LED light, (B) an LED sign, and (C) a rotating fan.

Table S1. Comparisons of areal density and electrochemical performance of the S-ZnO NRAs/Al and D-ZnO NRAs/Al hosts.

		S-ZnO NRAs/Al	D-ZnO NRAs/Al
Geometry Parameter	Areal Density (μm^{-2})	~150	~452
	Current Density (mA cm^{-2})	1	1
	Areal Capacity (mAh cm^{-2})	1	1
Electrochemical	Nucleation Overpotential (mV)	16.6	14.6
Performance	Cycle Number	760	760
	Polarization (mV)	16.3 - 17.7	16.0 - 17.5
	CE (%)	99.94	99.95

Table S2. Comparisons of Na deposition/dissolution performance of the D-ZnO NRAs/Al host tested at different current densities and areal capacities.

Current Density (mA cm^{-2})	Areal Capacity (mAh cm^{-2})	Cycle Number	Accumulated Capacity (mAh cm^{-2})	CE (%)
1	1	1500	1500	99.95
2	1	2670	2670	99.97
5	1	3600	3600	99.97
8	1	4950	4950	99.96
10	1	2955	2970	99.95
10	2	1560	3120	99.96
10	5	318	1590	99.93
10	8	180	1440	99.84
10	10	150	1500	99.59
10	20	100	2000	100

Table S3. Comparisons of electrochemical performance for the D-ZnO NRAs/Al and other hosts reported previously.

Electrode	Electrolyte	Current Density (mA cm^{-2})	Areal Capacity (mAh cm^{-2})	Cycles	Accumulated Capacity (mAh cm^{-2})	CE (%)	Ref.
CoP@N/P-CMFs	1M $NaPF_6$ in Diglyme	10	10	330	3300	99.97	1
SnNCNFs	1M $NaPF_6$ in Diglyme	3	3	2000	6000	99.96	2
Al-Cu@C	1M $NaPF_6$ in Diglyme	0.5	1	60	60	97.5	3
PC-CFe	1M $NaPF_6$ in Diglyme	10	10	500	5000	99.6	4
O-CCF	1M $NaPF_6$ in Diglyme	5	10	1000	10000	99.6	5
FCTF	1M $NaPF_6$ in Diglyme	2	1	400	400	99.6	6
NOCS	0.9 M $NaPF_6$ + 0.1 M $NaBF_4$ in Diglyme	2	2	1000	2000	99.97	7
MgF_2@NCHNFs	1M $NaPF_6$ in Diglyme	5	1	2000	2000	99.9	8
FMCNF	1M $NaPF_6$ in Diglyme	5	1	5000	5000	99.93	9
Cu_3P@Cu	1M $NaPF_6$ in Diglyme	4	4	250	1000	99.12	10
N-CSs/Cu	1M $NaPF_6$ in Diglyme	2	2	900	1800	99.99	11
Cu	1M $NaPF_6$ in Diglyme + SiO_2	1	2	500	1000	-	12
Cu	2.0 M $NaPF_6$ in DEE	1	1	150	150	99.89	13
Cu@Au	1M $NaSO_3CF_3$ in Diglyme	2	2	300	600	99.8	14
Bi-NAs@Cu	1M $NaPF_6$ in Diglyme	1	1	1000	1000	99.8	15
KJB@Cu	1M $NaPF_6$ in Diglyme	0.5	1	150	150	99.8	16
PBA@Cu	1M $NaPF_6$ in Diglyme	5	1	500	500	99.7	17
MRS-SbSA@Cu	1M $NaPF_6$ in DME	1	1	1800	1800	99.99	18
SnO_2@HMCNS @Cu	1M $NaPF_6$ in Diglyme	4	2	1800	3600	99.8	19
Ru/NC-Cu	1M $NaPF_6$ in Diglyme	1	1	1000	2000	100	20
Al	Na-SSZE	1	1	1000	1000	99.84	21
Porous Al	1M $NaPF_6$ in Diglyme	1	0.5	1000	500	99.8	22
Carbon/Al	1M $NaPF_6$ in Diglyme	0.5	0.25	1000	250	99.8	23

Electrode	Electrolyte	Current Density (mA cm^{-2})	Areal Capacity (mAh cm^{-2})	Cycles	Accumulated Capacity (mAh cm^{-2})	CE (%)	Ref.
3D Zn@Al	1 M $NaPF_6$ in DME	2	2	500	1000	99.5	24
p-Al@C	0.6 M NaOTF + 0.4 M $NaBF_4$ in Diglyme	0.5	0.5	100	50	99.88	25
BTO/C-Al	$Na_3Zr_2Si_2PO_{12}$	0.1	1	165	165	98.2	26
NbMoTaWV@Al	1 M $NaPF_6$ in DME	2	2	1000	2000	99.5	27
E-Zn@Al	1M $NaPF_6$ in Diglyme	3	3	1000	3000	99.95	28
Al/C	TGPE	0.5	0.5	400	200	99.92	29
SnCu@Al	1M $NaPF_6$ in Diglyme	1	1	500	500	99.95	30
Al(100)	1M $NaPF_6$ in DME	4	4	100	400	99.9	31
Al~HC	1M $NaPF_6$ in Diglyme	6	1	2500	2500	99.75	32
D-ZnO NRAs/Al	1M $NaPF_6$ in Diglyme	10	20	100	2000	100	This Work

Table S4. Comparisons of cycling performance of the D-ZnO NRAs/Al-Na anode with different current densities and areal capacities.

Depth-of-Discharge (%)	Current Density (mA cm^{-2})	Areal Capacity (mAh cm^{-2})	Cycle Number	Time (h)	Accumulated Capacity (mAh cm^{-2})
50	2	2	1000	2000	2000
50	5	5	1000	2000	5000
50	8	8	1000	2000	8000
50	10	10	1000	2000	10000
60	1	1	500	1000	500
70	1	1	150	300	150

Table S5. Comparisons of electrochemical performance of the D-ZnO NRAs/Al-Na and other composite Na anodes reported previously.

Electrode	Electrolyte	Current Density (mA cm^{-2})	Areal Capacity (mAh cm^{-2})	Depth-of-Discharge (%)	Accumulated Capacity (mAh cm^{-2})	Life (h)	Ref.
CoP@N/P-CMFs-Na	1M $NaPF_6$ in Diglyme	8	8	50	3200	800	1
SnNCNFs	1M $NaPF_6$ in Diglyme	10	10	86	3500	700	2
PC-CFe-Na	1M $NaPF_6$ in Diglyme	5	5	-	4125	1650	4
O-CCF-Na	1M $NaPF_6$ in Diglyme	50	1	16.67	10000	400	5
FCTF-Na	1M $NaPF_6$ in Diglyme	2	2	100	140	140	6
NOCS-Na	0.9 M $NaPF_6$ + 0.1 M $NaBF_4$ in Diglyme	0.5	1	50	250	1000	7
MgF_2@NCHNFs-Na	1M $NaPF_6$ in Diglyme	5	2	16.7	9000	3600	8
FMCNF-Na	1M $NaPF_6$ in Diglyme	10	10	94	10000	2000	9
Cu_3P@Cu-Na	1M $NaPF_6$ in Diglyme	2	2	-	2000	2000	10
N-CSs-Na	1M $NaPF_6$ in Diglyme	2	2	-	1500	1500	11
Bi-NAs@Cu-Na	1M $NaPF_6$ in Diglyme	1	3	75	600	1200	15
PBA@Cu-Na	1M $NaPF_6$ in Diglyme	5	1	66.67	1500	600	17
MRS-SbSA@Cu-Na	1M $NaPF_6$ in DME	2	2	91	2000	2000	18
SnO_2@HMCNS@Cu-Na	1M $NaPF_6$ in Diglyme	8	4	-	2800	700	19
Porous Al-Na	1M $NaPF_6$ in Diglyme	0.5	0.5	25	250	1000	22
3D Zn@Al-Na	1 M $NaPF_6$ in DME	2	1	50	1500	1500	24
BTO/C-Al-Na	$Na_3Zr_2Si_2PO_{12}$	0.5	0.5	-	125	500	26
NbMoTaWV@Al	1 M $NaPF_6$ in DME	2	1	50	1000	1000	27
E-Zn@Al-Na	1M $NaPF_6$ in Diglyme	3	3	-	1500	1000	28
Al/C-Na	TGPE	1	1	-	300	600	29
SnCu@Al-Na	1M $NaPF_6$ in Diglyme	5	5	80	3750	1500	30
Al(100)-Na	1M $NaPF_6$ in DME	1	1	-	750	1500	31
NST-Na	1M $NaPF_6$ in Diglyme	2	10	60	1500	1500	33

Electrode	Electrolyte	Current Density (mA cm^{-2})	Areal Capacity (mAh cm^{-2})	Depth-of-Discharge (%)	Accumulated Capacity (mAh cm^{-2})	Life (h)	Ref.
F-A-Al-Na	Na[FSA] in $[C_2C_1im][FSA]$	0.5	0.5	25	150	600	34
HCOONa-Na	1M $NaPF_6$ in Diglyme	2	1	-	2200	2200	35
D-ZnO NRAs/Al-Na	1M $NaPF_6$ in Diglyme	10	10	50	10000	2000	This Work

Table S6. Comparisons of electrochemical performance of the anode-less D-ZnO NRAs/Al-Na//NVOPF cell with previously reported works.

Anode	Cathode	Mass Loading (mg cm^{-2})	N/P Ratio	Voltage (V)	Current Density (mA g^{-1})	Capacity (mAh g^{-1})	Cycle Number	Capacity Retention (%)	Ref.
SnNCNF-Na	S@pPAN	10	1.5	0.6-2.8	750	-	80	81	2
FCTF-Na	NVP	6.4	1.5	2.5-3.8	236	108.9	300	94.7	6
NOCS-Na	NVP	11.4	1.5	2.0-3.8	500	100	800	96.2	7
FMCNF-Na	NVP	12	2.1	2.5-3.8	234	-	600	91	9
N-CSs	NNMO	4	-	2.5-3.9	-	-	200	86.3	11
Bi-NAs@Cu-Na	NTP	6	1	1.5-2.5	133	95.18	267	93.22	15
KJB@Cu-Na	NVP	-	-	2.5-3.8	60	-	150	94.16	16
PBA@Cu-Na	NVP	3	3	2.2-3.8	590	-	1500	94.3	17
E-Zn@Al-Na	NVP	10	1.5	2.5-3.8	117	-	600	99	28
SnCu@Al-Na	NVP	4.5	1	2.5-3.8	117	-	400	93	30
D-ZnO NRAs/Al-Na	NVOPF	32.26	1.5	2.0-4.3	130	102.5	105	87.1	This Work

Table S7. Comparisons of electrochemical performance of the anode-free D-ZnO NRAs/Al//NVOPF cell with previously reported works.

Anodic Current Collector	Cathode	Mass Loading (mg cm^{-2})	Voltage (V)	Current Density (mA g^{-1})	Capacity (mAh g^{-1})	Cycle Number	Capacity Retention (%)	Ref.
CoP@N/P-CMFs	NVP	18	2.0-3.0	117	82.7	120	87.38	1
SnNCNF	NVP	10	2.6-3.8	234	89.3	80	89	2
Al-Cu@C	NVP/C	3.5-4.0	1.5-3.8	120	-	50	-	3
PC-CFe	NVP	10	2.6-3.8	1 mA cm^{-2}	103	100	97	4
O-CCF	NVP	11	2.5-4.0	1 mA cm^{-2}	103.7	100	96	5
FCTF	NVP	7.4	2.5-3.8	240	-	400	56	6
NOCS	NVP	12.3	2.0-3.8	200	81.4	350	79.0	7
MgF_2@NCH NFs	NVP	8	2.5-3.8	236	-	50	91.2	8
FMCNF	NVP	20	2.5-3.8	35.1	-	200	90	9
Cu_3P@Cu	NVP	13.6	2.5-3.5	60	76.1	75	-	10
N-CPs	NNMO	4	2.5-3.8	50	-	200	86.3	11
Cu	NVP	4	2.6-3.8	0.5 mA cm^{-2}	71.88	100	75	12
Cu	NFM	4.5	2.0-3.8	70	-	100	82.3	13
Cu@Au	Na-FeS_2	-	0.8-3.0	-	140	50	-	14
PBA@Cu-Na	NVP	3	2.2-3.8	590	-	300	75.29	17
MRS-SbSA@Cu	NVP	7	2.0-3.6	117	-	100	83	18
SnO_2@HMC NS@Cu	NVP	3.5	2.8-4.0	100	-	200	79.5	19
Ru/NC-Cu	NVP	3	2.5-3.6	50	-	100	98.1	20
C@Al	NVPOF	7.1	2.0-4.25	0.49 mA cm^{-2}	97.9	370	89.2	21
Porous Al	Na-TiS_2	-	1.3-2.6	0.1 mA cm^{-2}	~160	200	~64	22
Carbon/Al	$Na_{1.5}FeS_2$	5	0.8-3.0	0.125 mA cm^{-2}	335	40	-	23
3D Zn@Al	NVP	1.8	2.5-3.8	59	-	100	98.8	24
BTO/C-Al	NVP	-	2.5-3.8	0.1 mA cm^{-2}	104.1	300	95.5	26

Anodic Current Collector	Cathode	Mass Loading (mg cm^{-2})	Voltage (V)	Current Density (mA g^{-1})	Capacity (mAh g^{-1})	Cycle Number	Capacity Retention (%)	Ref.
NbMoTaWV @Al	NVP	1.8	2.5-3.8	-	-	300	96.5	27
E-Zn@Al	NVP	32	2.5-3.8	3.74 mA cm^{-2}	103	90	86	28
Al/C	NVP	10	2.5-3.8	117	-	500	79	29
SnCu@Al	NVP	4.6	2.5-3.8	117	64.5	200	71	30
Al(100)	NVP	1.5	2.5-3.6	1.755 mA cm^{-2}	68	100	-	31
Al~HC	NFPP	5	1.5-3.5	20	84.6	100	90.5	32
NST	NVP	2.5	3.2-3.6	118	73.15	100	77	33
F-A-Al	NVP	11.3	2.4-3.6	35.1	-	50	46.1	34
SF-Cu	NVP	10	2.8-3.7	58.5	86.3	400	88	35
C@Al	NNCFM	8.52	2.0-4.0	30	63.9	250	-	36
GC@Al	NCNFM	15.12	2.0-3.8	63.5	-	260	84	37
D-ZnO NRAs/Al	NVOPF	5.1	2.0-4.3	65	104.2	105	86	This Work

Supplementary References

1. An, Y.; Pei, Z.; Luan, D.; Lou, X. W. Foldable Anode-Free Sodium Batteries Enabled by N,P-Codoped Carbon Macroporous Fibers Incorporated with CoP Nanoparticles. *Sci. Adv.* **2025,** *11*, eadv2007.

2. Li, S.; Zhu, H.; Liu, Y.; Wu, Q.; Cheng, S.; Xie, J. Space-Confined Guest Synthesis to Fabricate Sn-Monodispersed N-Doped Mesoporous Host Toward Anode-Free Na Batteries. *Adv. Mater.* **2023,** *35*, 2301967.

3. Li, H.; Zhang, H.; Wu, F.; Zarrabeitia, M.; Geiger, D.; Kaiser, U.; Varzi, A.; Passerini, S. Sodiophilic Current Collectors Based on MOF-Derived Nanocomposites for Anode-Less Na-Metal Batteries. *Adv. Energy Mater.* **2022,** *12*, 2202293.

4. Lee, K.; Lee, Y. J.; Lee, M. J.; Han, J.; Lim, J.; Ryu, K.; Yoon, H.; Kim, B. H.; Kim, B. J.; Lee, S. W. A 3D Hierarchical Host with Enhanced Sodiophilicity Enabling Anode-Free Sodium-Metal Batteries. *Adv. Mater.* **2022,** *34*, 2109767.

5. Li, T.; Sun, J.; Gao, S.; Xiao, B.; Cheng, J.; Zhou, Y.; Sun, X.; Jiang, F.; Yan, Z.; Xiong, S. Superior Sodium Metal Anodes Enabled by Sodiophilic Carbonized Coconut Framework with 3D Tubular Structure. *Adv. Energy Mater.* **2020,** *11*, 2003699.

6. Zhuang, R.; Zhang, X.; Qu, C.; Xu, X.; Yang, J.; Ye, Q.; Liu, Z.; Kaskel, S.; Xu, F.; Wang, H. Fluorinated Porous Frameworks Enable Robust Anode-Less Sodium Metal Batteries. *Sci. Adv.* **2023,** *9*, eadh8060.

7. Zhang, R.; Zhu, X.; Xie, T.; Jiang, C.; Ma, J.; Xie, C.; Ji, H.; Wang, J.; Li, H.; Wang, H. N,O Co-Doped Carbon Spheres Enable Stable Anode-Less Sodium Metal Batteries. *Small Methods* **2025,** *9*, 2401884.

8. Guo, W.; Liu, X.; Mu, Y.; Yue, G.; Liu, J.; Zhu, K.; Cui, Z.; Wang, N.; Chen, Z.; Zhao, Y. Outside-in Directional Sodium Deposition Through Self-Supporting Gradient Fluorinated Magnesium Alloy Framework Toward High-Rate Anode-Free Na Batteries. *Energy Storage Mater.* **2024,** *73*, 103840.

9. Zhu, H.; Peng, L.; Wu, J.; Li, S.; Wu, Q.; Cheng, S.; Xie, J.; Lu, J. Fluorine-Doped Micropore-Covered Mesoporous Carbon Nanofibers for Long-Lasting Anode-Free Sodium Metal Batteries. *Nat. Commun.* **2025,** *16*, 5494.

10. Zhang, W.; Zheng, J.; Ren, Z.; Wang, J.; Luo, J.; Wang, Y.; Tao, X.; Liu, T. Anode-Free Sodium Metal Pouch Cell Using Cu_3P Nanowires In Situ Grown on Current Collector. *Adv. Mater.* **2024,** *36*, 2310347.

11. Huang, B.; Sun, S.; Wan, J.; Zhang, W.; Liu, S.; Zhang, J.; Yan, F.; Liu, Y.; Xu, J.; Cheng, F.; Xu, Y.; Lin, Y.; Fang, C.; Han, J.; Huang, Y. Ultrahigh Nitrogen Content Carbon Nanosheets for High Stable Sodium Metal Anodes. *Adv. Sci.* **2023,** *10*, 2206845.

12. Panchal, R. A.; Datta, J.; Varude, V.; Bhimani, K.; Mahajani, V.; Kamble, M.; Anjan, A.; Manoj, R. M.; Zha, R. H.; Datta, D.; Koratkar, N. Nano-Silica Electrolyte Additive Enables Dendrite Suppression in an Anode-Free Sodium Metal Battery. *Nano Energy* **2024,** *129*, 110010.

13. Zou, Y.; Zhang, B.; Luo, H.; Yu, X.; Yang, M.; Zheng, Q.; Wang, J.; Jiao, C.; Chen, Y.; Zhang, H.; Xue, J.; Kuai, X.; Liao, H. G.; Ouyang, C.; Ning, Z.; Qiao, Y.; Sun, S. G. Electrolyte Solvation Engineering Stabilizing Anode-Free Sodium Metal Battery With 4.0 V-Class Layered Oxide Cathode. *Adv. Mater.* **2024,** *36*, 2410261.

14. Tang, S.; Qiu, Z.; Wang, X.-Y.; Gu, Y.; Zhang, X.-G.; Wang, W.-W.; Yan, J.-W.; Zheng, M.-S.; Dong, Q.-F.; Mao, B.-W. A Room-Temperature Sodium Metal Anode Enabled by a Sodiophilic Layer. *Nano Energy* **2018,** *48*, 101-106.

15. Bai, Y.; Zheng, X.; Liu, H.; Huang, J.; Zhang, L.; Otitoju, T. A.; Sun, T.; Liu, H. K.; Dou, S. X.; Wu, C. Honeycomb-Like Superstructure of 3D Sodiophilic Host for Anode-Free Sodium Batteries. *Energy Storage Mater.* **2025,** *74*, 103926.

16. Kang, S.; Geng, F.; Li, Z.; Jiang, Y.; Shen, M.; Chen, Q.; Lou, X.; Hu, B. Progressive Self-Leveling Deposition Improves the Cyclability of Anode-less Sodium Metal Batteries Revealed by In Situ EPR Imaging. *ACS Energy Lett.* **2024,** *9*, 1633-1638.

17. Hu, Z.; Liu, L.; Wang, X.; Lu, H.; Zheng, Q.; Gao, Y.; Wang, J.; Qi, Y.; Han, C.; Li, W. In Situ Integration of Rapid Ion-Diffusion Interlayers on Cu Current Collectors toward Ultrafast Anode-Free Sodium Metal Batteries. *ACS Nano* **2025,** *19*, 23193-23208.

18. Zhao, S.; Chen, X.; Wang, Y.; Hong, Z.; Zheng, L.; Zhang, Y.; Wei, M.; Lu, J. Highly Reversible Sodium Metal Batteries Enabled by Extraordinary Alloying Reaction ofSingle-Atom Antimony. *Adv. Energy Mater.* **2024,** *15*, 2403432.

19. Zhu, H.; Yan, Z.; Wang, X.; Liu, W.; Guo, L.; Wei, Q.; Wang, M. S. Synergistic Effect of Mesoporous Carbon-Based Framework with Sodiophilic Nanoparticles for Stable Sodium Metal Anodes. *Adv. Funct. Mater.* **2025,** *35*, 2502032.

20. Hao, C.; Zhang, X.; He, Z.; Gao, M.; Liu, Y.; Pan, H.; Sun, W. Robust Solid Electrolyte Interphase Engineered by Catalysis Chemistry Toward Durable Anode-Free Sodium Metal Batteries. *Angew. Chem. Int. Ed.* **2025,** *64*, e202503691.

21. Lu, Z.; Yang, H.; Wu, G.; Shan, P.; Lin, H.; He, P.; Zhao, J.; Yang, Y.; Zhou, H. A "Liquid-In-Solid" Electrolyte for High-Voltage Anode-Free Rechargeable Sodium Batteries. *Adv. Mater.* **2024,** *36*, 2404569.

22. Liu, S.; Tang, S.; Zhang, X.; Wang, A.; Yang, Q. H.; Luo, J. Porous Al Current Collector for Dendrite-Free Na Metal Anodes. *Nano Lett.* **2017,** *17*, 5862-5868.

23. Cohn, A. P.; Muralidharan, N.; Carter, R.; Share, K.; Pint, C. L. Anode-Free Sodium Battery Through In Situ Plating of Sodium Metal. *Nano Lett.* **2017,** *17*, 1296-1301.

24. Cai, Z.; Tang, F.; Yang, Y.; Xu, S.; Xu, C.; Liu, L.; Rui, X. A Multifunctional Super‐Sodiophilic Coating on Aluminum Current Collector for High-Performance Anode-Free Na-Metal Batteries. *Nano Energy* **2023,** *116*, 108814.

25. Zhu, Q.; Yu, D.; Chen, J.; Cheng, L.; Tang, M.; Wang, Y.; Li, Y.; Yang, J.; Wang, H. A 110 Wh kg^{-1} Ah-Level Anode-Free Sodium Battery at -40°C. *Joule* **2024,** *8*, 482-495.

26. Sun, C.; Li, Y.; Sun, Z.; Yuan, X.; Jin, H.; Zhao, Y. Ferroelectric Interface for Efficient Sodium Metal Cycling in Anode-Free Solid-State Batteries. *Mater. Today* **2024,** *80*, 395-405.

27. Liu, L.; Cai, Z.; Yang, S.; Yang, Y.; Yao, Y.; He, S.; Xu, S.; Wu, Z.; Pan, H.; Rui, X.; Yu, Y. Multifunctional High-Entropy Alloy Nanolayer Toward Long-Life Anode-Free Sodium Metal Battery. *Adv. Mater.* **2025,** *37*, 2413331.

28. Ge, J.; Ma, C.; Zhang, Y.; Ma, P.; Zhang, J.; Xie, Z.; Wen, L.; Tang, G.; Wang, Q.; Li, W.; Guo, X.; Guo, Y.; Zhang, E.; Zhang, Y.; Zhao, L.; Chen, W. Edge Electron Effect Induced High-Entropy SEI for Durable Anode-Free Sodium Batteries. *Adv. Mater.* **2025**, *37*, 2413253.

29. Xu, Z.; Lin, C.; Qiu, J.; Wang, Z. Polymer-Regulated Solvation and Interphase Engineering for Long-Life and Safe Quasi-Solid-State Anode-Free Sodium Batteries. *Adv. Mater.* **2025,** *37*, 2506037.

30. Shi, J.; Wang, D.; Liu, Q.; Yu, Z.; Huang, J. Q.; Zhang, B. Intermetallic Layers with Tuned Na Nucleation and Transport for Anode-Free Sodium Metal Batteries. *Nano Lett.* **2025,** *25*, 1800-1807.

31. Tang, F.; Yang, Y.; Liu, C.; Yang, S.; Xu, S.; Yao, Y.; Yang, H.; Yang, Y.; He, S.; Pan, H.; Rui, X.; Yu, Y. Initially Anode-Free Sodium Metal Battery Enabled by Strain-Engineered Single-Crystal Aluminum Substrate with (100)-Preferred Orientation. *Nat. Commun.* **2025,** *16*, 2280.

32. Ruan, J.; Hu, J.; Li, Q.; Luo, S.; Yang, J.; Liu, Y.; Song, Y.; Zheng, S.; Sun, D.; Fang, F.; Wang, F. Current Collector Interphase Design for High-Energy and Stable Anode-Less Sodium Batteries. *Nat. Sustain.* **2025,** *8*, 530-541.

33. Wang, Y.; Dong, H.; Katyal, N.; Hao, H.; Liu, P.; Celio, H.; Henkelman, G.; Watt, J.; Mitlin, D. A Sodium-Antimony-Telluride Intermetallic Allows Sodium-Metal Cycling at 100% Depth of Discharge and as an Anode-Free Metal Battery. *Adv. Mater.* **2022,** *34*, 2106005.

34. Wu, S.; Hwang, J.; Matsumoto, K.; Hagiwara, R. The Rational Design of Low-Barrier Fluorinated Aluminum Substrates for Anode-Free Sodium Metal Battery. *Adv. Energy Mater.* **2023,** *13*, 2302468.

35. Wang, C.; Zheng, Y.; Chen, Z. N.; Zhang, R.; He, W.; Li, K.; Yan, S.; Cui, J.; Fang, X.; Yan, J.; Xu, G.; Peng, D.; Ren, B.; Zheng, N. Robust Anode-Free Sodium Metal Batteries Enabled by Artificial Sodium Formate Interface. *Adv. Energy Mater.* **2023,** *13*, 2204125.

36. Lu, Z.; Yang, H.; Yang, Q. H.; He, P.; Zhou, H. Building a Beyond Concentrated Electrolyte for High-Voltage Anode-Free Rechargeable Sodium Batteries. *Angew. Chem. Int. Ed.* **2022,** *61*, e202200410.

37. Li, Y.; Zhou, Q.; Weng, S.; Ding, F.; Qi, X.; Lu, J.; Li, Y.; Zhang, X.; Rong, X.; Lu, Y.; Wang, X.; Xiao, R.; Li, H.; Huang, X.; Chen, L.; Hu, Y.-S. Interfacial Engineering to Achieve an Energy Density of over 200 Wh kg^{-1} in Sodium Batteries. *Nat. Energy* **2022,** *7*, 511-519.
